# Signatures of intramolecular vibrational and vibronic Q$_{\mathrm{x}}$–Q$_{\mathrm{y}}$ coupling effects in absorption and CD spectra of chlorophyll dimers

**DOI:** 10.1007/s11120-022-00946-3

**Published:** 2022-08-30

**Authors:** Joachim Seibt, Dominik Lindorfer, Thomas Renger

**Affiliations:** Institut für Theoretische Physik, Johannes Kepler Universität Linz, Altenberger Str. 69, 4040 Linz, Austria

**Keywords:** WSCP, Absorption, Circular dichroism

## Abstract

An electron-vibrational coupling model that includes the vibronic (non-adiabatic) coupling between the Q$_{\mathrm{y}}$ and Q$_{\mathrm{x}}$ transitions of chlorophyll (Chl), created by Reimers and coworkers (Scientific Rep. 3, 2761, 2013) is extended here to chlorophyll dimers with interchlorophyll excitonic coupling. The model is applied to a Chl *a* dimer of the water-soluble chlorophyll binding protein (WSCP). As for isolated chlorophyll, the vibronic coupling is found to have a strong influence on the high-frequency vibrational sideband in the absorption spectrum, giving rise to a band splitting. In contrast, in the CD spectrum the interplay of vibronic coupling and static disorder leads to a strong suppression of the vibrational sideband in excellent agreement with the experimental data. The conservative nature of the CD spectrum in the low-energy region is found to be caused by a delicate balance of the intermonomer excitonic coupling between the purely electronic Q$_{\mathrm{y}}$ transition and the Q$_{\mathrm{y}}$ transition involving intramolecular vibrational excitations on one hand and the coupling to higher-energy electronic transitions on the other hand.

**Supplementary Information:**

The online version contains supplementary material available at 10.1007/s11120-022-00946-3.

## Introduction

Non-adiabatic couplings between different excited electronic states of chlorophyll (Chl) and related pigments in photosynthetic antennae give rise to a fast internal conversion of excitation energy to the first excited state S$_1$, from where excitation energy transfer to the photosynthetic reaction center (RC) starts. In this way, the absorption spectrum of the RC is increased spectrally and spatially. Internal conversion timescales in the 100 fs range have been reported both experimentally, using time-resolve spectroscopy (Shi et al. 2005; Bricker et al. 2015; Meneghin et al. 2017; Song et al. 2019; Do et al. 2022), and theoretically from non-adiabatic excited state molecular dynamics simulations (Bricker et al. 2015; Zheng et al. 2017; Fortino et al. 2021).

For the two lowest excited states, S$_1$ and S$_2$, the non-adiabatic coupling is so strong that a quantum mechanic mixing between the $\mathrm S_0 \rightarrow \mathrm S_1$ (termed Q$_{\mathrm{y}}$) and the $\mathrm S_0 \rightarrow \mathrm S_2$ (termed Q$_{\mathrm{x}}$) transitions can occur. This mixing is particularly strong if the 0-1 transition of Q$_{\mathrm{y}}$ and the 0-0 transition of Q$_{\mathrm{x}}$ are in near resonance. Here, “0-1” refers to an electronic transition that is accompanied by the excitation of the first excited state of a high-frequency intramolecular vibrational mode and “0-0” is a purely electronic transition. Such a resonance condition is met, e.g., in Chl *a* and to a much lesser extent in bacteriochlorophyll *a* (Reimers et al. 2013).

**Fig. 1:**
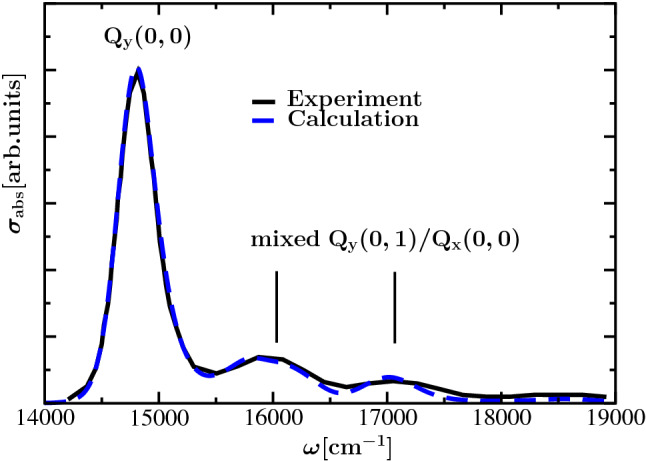
Comparison between experimental absorption spectrum of Chl *a* in ether (Reimers et al. 2013) (black solid line) at $T=300 \hbox {K}$ and calculation (blue dashed line) using a vibronic coupling model adopted from Reimers et al. (2013), as described in the theory part. In the calculation a 0-0 transition energy of $14910\,{\hbox {cm}^{-1}}$ ($670.7{\hbox {nm}}$) of Chl *a* was assumed

In a landmark study, Reimers and coworkers (Reimers et al. 2013) provided compelling evidence for this mixing and thereby solved a long-standing puzzle concerning the assignment of the Q$_{\mathrm{y}}$ vibrational sideband and the Q$_{\mathrm{x}}$ transition (see Fig. 1). Using a simple model that includes a non-adiabatic coupling between the S$_1$ and S$_2$ states, which is assumed to be linear in the coordinate of a vibronic coupling mode, and a remaining set of intramolecular modes that couple separately to the Q$_{\mathrm{y}}$ and Q$_{\mathrm{x}}$ transitions, they were able to explain the absorption, fluorescence, and magnetic circular dichroism spectra of 32 different chlorophyllides in various environments. We follow Reimers *et al.* and will call this non-adiabatic mixing between Q$_{\mathrm{y}}$ and Q$_{\mathrm{x}}$ “vibronic coupling”. Independent evidence for this vibronic coupling has been reported from polarized two-dimensional electronic spectroscopy on Chl *a* (Song et al. 2019) and Chl *c* (Bukartė et al. 2020) in solution.

In the present study, the vibronic coupling model of Reimers et al. (2013) is extended to describe optical spectra of chlorophyll dimers with strong excitonic coupling. A suitable model system to study the interplay of intrachromophore vibronic and interchromophore excitonic couplings is the water-soluble chlorophyll binding protein (WSCP). It is a tetramer with quasi D2 symmetry (Horigome et al. 2007; Bednarczyk et al. 2016) containing 4 chlorophyll *a* pigments that are arranged in two dimers with strong intra- and weak inter-dimer excitonic couplings. WSCP has been used as a simple model system for the study of pigment-pigment and pigment-protein interactions (Hughes et al. 2006; Renger et al. 2007; Adolphs et al. 2016; Friedl et al. 2022; Pieper et al. 2011a, b; Alster et al. 2014; Rosnik and Curutchet 2015; Bednarczyk et al. 2016; Agostini et al. 2018; Palm et al. 2018; Prabahar et al. 2020; Fresch et al. 2020; Lahav et al. 2021). Due to their quasi-symmetric protein environment, all four Chls have the same mean local transition energy (site energy). Another simplifying aspect of WSCP is the fact that wavefunction overlap between the Chls is sufficiently low, such that short-range contribution to the site energies and excitonic couplings can safely be neglected. On the other hand, the Chls in the dimers are close enough for strong excitonic coupling, giving rise to a delocalization of excited states. The latter leads to a strong redistribution of oscillator strength between the two lowest exciton states of the dimer (Hughes et al. 2006; Renger et al. 2007). Due to the open sandwich geometry of local transition dipole moments, the high-energy exciton state gets most of the oscillator strength of the Q$_{\mathrm{y}}$ 0-0 transitions of the two Chls.

**Fig. 2:**
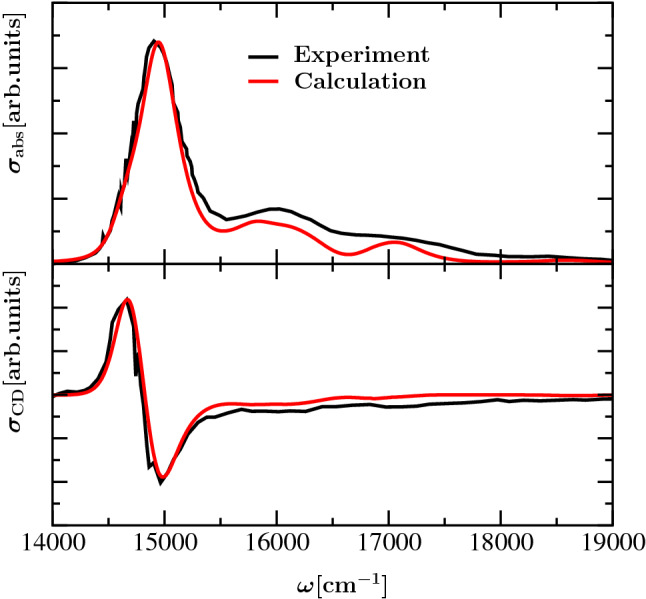
Comparison between experimental absorption (upper part) and circular dichroism (lower part) spectra of Chl *a* WSCP measured at $T=300\,{\hbox {K}}$ (Palm et al. 2017) and calculations using a dimer exciton model with vibronic coupling described in the theory part. In the calculation a local transition energy of $14940\,{\hbox {cm}^{-1}}$ ($669.3{\hbox {nm}}$) of Chl *a* has been assumed

Whereas this low-energy region of the optical spectra of WSCP has been thoroughly investigated (Hughes et al. 2006; Renger et al. 2007; Dinh and Renger 2015, 2016; Adolphs et al. 2016; Friedl et al. 2022; Pieper et al. 2011b), the spectrum at higher energies is less understood theoretically. In this region, we expect a mixing between the Q$_{\mathrm{x}}$ and Q$_{\mathrm{y}}$ transitions of the Chls in the dimer that are coupled vibronically in the monomers and excitonically between the monomers. Interestingly, there are relatively strong experimental signals in the absorption spectrum at higher energies, but barely any signals in the circular dichroism (CD) spectrum (see Fig. 2). Since the intrinsic CD of Chls is small, the CD spectrum is determined by exciton contributions that vanish for localized excited states. In contrast, in the absorption spectrum the excitonic coupling can only redistribute the oscillator strength without changing the overall intensity of the spectrum. Hence, our working hypothesis for the present study is that a localization of excited states involving excited intramolecular vibrational modes occurs that leads to a suppression of the CD signal at high energies.

The remaining parts of this work are organized as follows: We start by summarizing the monomer Hamiltonian of Reimers et al. (2013) containing the vibronic coupling between the Q$_{\mathrm{y}}$ and Q$_{\mathrm{x}}$ transitions. Next, this Hamiltonian is extended by introducing the intermonomer excitonic coupling. Afterward, the low-frequency part of the spectral density of the local exciton-vibrational coupling of the monomers is used to introduce a system-bath interaction Hamiltonian that is applied afterward to derive line-shape functions of optical transitions. Next, we summarize the parameterization of the monomer and dimer Hamiltonians that are applied to describe optical spectra of isolated Chl *a* in ether and of a Chl *a* dimer in WSCP. Our theoretical analysis comprises a step-by-step investigation of the influence of different parts of the Hamiltonian on the optical spectra presented in Figs. 1 and 2. Finally we summarize our findings concerning the structure of the Q$_{\mathrm{y}}$–Q$_{\mathrm{x}}$ vibrational side band in the absorption spectrum, the suppression of this band in the CD spectrum and the conservative nature of the latter.

## Theory

### Monomer Hamiltonian

Based on the model proposed in Reimers et al. (2013), we start from the Hamiltonian of the monomer with electronic ground state $\mid \mathrm{S_0} \rangle$ and singly excited states $\mid m \rangle \in \{ \mid \mathrm{S_1} \rangle , \mid \mathrm{S_2} \rangle , \mid \mathrm{S_3} \rangle , \mid \mathrm{S_4} \rangle \}$. While the states $\mid \mathrm{S_1} \rangle$ and $\mid \mathrm{S_2} \rangle$ are vibronically coupled, this is not the case for the states $\mid \mathrm{S_3} \rangle$ and $\mid \mathrm{S_4} \rangle$, which are taken into account additionally compared to Reimers et al. (2013). Electronic transitions from $\mid \mathrm{S_0} \rangle$ to $\mid \mathrm{S_3} \rangle$ and to $\mid \mathrm{S_4} \rangle$, denoted as $\mathrm{B_y}$ and $\mathrm{B_x}$, respectively, are distinct components of the Soret band and have been characterized by quantum chemical calculations in an earlier work (Lindorfer et al. 2017). For simplicity they are assumed to be purely electronic states without vibrational substructure in our model. The corresponding eigenenergies are $\epsilon _0= 0$ and $\epsilon _m$. Electronic excitation from $\mid 0 \rangle$ to $\mid m \rangle$ goes along with displacement of the potentials of $N_{m}$ vibrational oscillator modes, where each of them is addressed by the index *i*. We denote their respective vibrational frequencies, position coordinate operators, displacements, and momentum operators as $\omega _{i}$, $\hat{q}_{i}$, $d_{m,i}$ and $\hat{p}_{i}$, respectively. While we assume that the displacement of each vibrational mode depends on the electronic state, the vibrational frequency is assumed to be equal in all electronic states. Furthermore, we separately specify the corresponding parameters and operators of the vibronic coupling mode as $\omega _{\mathrm{VC}}$, $\hat{q}_{\mathrm{VC}}$, $d_{\mathrm{VC}}$ and $\hat{p}_{\mathrm{VC}}$, which are all independent of the electronic state. The vibronic coupling mode can be associated with the coupling mode of a conical intersection, while among the further vibrational modes at least those with different Huang–Rhys factors in Q_y_ and Q_x_ excitation share the properties of tuning modes (Robb 2011). Different from Reimers et al. (2013), we do not apply a scaling of the position coordinate by the square root of the inverse vibrational frequency, $\tilde{q}=\sqrt{\frac{1}{\omega }} q$, so that $\frac{h \nu }{2} \tilde{q}^2=\frac{\hbar \omega }{2} \tilde{q}^2$ in Reimers et al. (2013) can be identified with $\frac{\hbar \omega }{2} \omega ^2 q^2$. We furthermore set $\hbar = 1$. The scaling of the vibrational coordinate also influences the vibronic coupling constant in terms of $\tilde{\alpha }_{\mathrm{VC}}=\sqrt{\frac{1}{\omega _{\mathrm{VC}}}} \alpha _{\mathrm{VC}}$. Altogether the monomer Hamiltonian can be written as

1 $$\begin{aligned} \begin{aligned} \hat{H}_{\mathrm{M}}&=\left[ \sum _i^{N_m} \frac{1}{2}(\omega _{i}^2 \hat{q}_{i}^2 +\hat{p}_{i}^2) +\frac{1}{2}(\omega _{\mathrm{VC}}^2 \hat{q}_{\mathrm{VC}}^2 +\hat{p}_{\mathrm{VC}}^2) \right] \mid 0 \rangle \langle 0 \mid \\&\quad +\sum _{m \in \{ \mathrm{S_1},\mathrm{S_2},\mathrm{S_3},\mathrm{S_4} \}} \left[ \epsilon _m +\sum _i^{N_m} \frac{1}{2}(\omega _{i}^2 (\hat{q}_{i}-d_{m,i})^2 +\hat{p}_{i}^2) \right. \\&\quad \left. +\frac{1}{2}(\omega _{\mathrm{VC}}^2 \hat{q}_{\mathrm{VC}}^2 +\hat{p}_{\mathrm{VC}}^2) \right] \mid m \rangle \langle m \mid \\&\quad +\alpha _{\mathrm{VC}} q_{\mathrm{VC}} (\mid \mathrm{S_1} \rangle \langle \mathrm{S_2} \mid +\mid \mathrm{S_2} \rangle \langle \mathrm{S_1} \mid ). \end{aligned} \end{aligned}$$

Please note that $d_{m,i}=0$ for $m \in \{ \mathrm{S_3},\mathrm{S_4} \}$, that is we do not take into account vibrational excitations of the $\mathrm{B_y} (\mathrm{S_0} \rightarrow \mathrm{S_3})$ and $\mathrm{B_x} (\mathrm{S_0} \rightarrow \mathrm{S_4})$ transitions. We select the vibronic coupling mode and the high-frequency intramolecular vibrational oscillators to be treated as part of the relevant system, while the low-frequency intermolecular ones enter in terms of contributions to an environment. Note that the harmonic oscillator potentials of the vibronic coupling mode in the diagonal elements are not displaced, but the linear dependence of the off-diagonal elements of the above Hamiltonian on the position operator of the vibronic coupling mode leads to an effective displacement $d_{\mathrm{VC, eff}}$ and accordingly to an effective Huang–Rhys factor $S_{\mathrm{VC, eff}}=\frac{1}{2} \omega _{\mathrm{VC}} d_{\mathrm{VC, eff}}^2$ if a diagonalization is applied. The basis representation of the monomer Hamiltonian is described in Appendix A. While this basis representation is formulated without restriction to a certain number of vibrational excitation quanta, we only take a small number of the possible vibrational excitations into account, as described in more detail in “Model assumptions and parameters” Section. However, we want to mention explicitly that simultaneous excitation of an intramolecular vibrational mode and a vibronic coupling mode are accounted for in our model.

### Dimer Hamiltonian

The Hamiltonian of an excitonic dimer is composed of Hamiltonians of the monomer units given in Eq. (1), which in the following will be referred to by introducing an additional superscript index, and excitonic coupling contributions between electronic transitions of the different monomer units. Thus, it is of the form

2 $$\begin{aligned} \hat{H}_{\mathrm{D}}= \sum _{\bar{a}} \hat{H}_{\mathrm{M}}^{(\bar{a})} +\sum _{\bar{a}} \sum _{\bar{b}} \sum _{\bar{m}} \sum _{\bar{n}} J^{(\bar{a}) (\bar{b})}_{\bar{m} \bar{n}} \mid \bar{m}^{(\bar{a})} \rangle \mid 0^{(\bar{b})} \rangle \langle 0^{(\bar{a})} \mid \langle \bar{n}^{(\bar{b})} \mid , \end{aligned}$$

where $\bar{a}$ and $\bar{b}$ count the pigments and $\bar{m}$ and $\bar{n}$ the electronic states of pigment $\bar{a}$ and $\bar{b}$, respectively. For the basis representation we use a product of the monomer bases with one of the monomer units being excited and the other one being de-excited (i.e., in the electronic ground state) in the formulation of singly excited states of the dimer. To reduce the size of the basis, we apply the so-called one-particle approximation (OPA) (Spano 2002, 2010; Hestand and Spano 2018) and only take a single product state composed of the lowest vibrational eigenfunctions of the explicitly treated intramolecular vibrational modes into account if the respective monomer unit is de-excited, while the complementary monomer unit in the excited electronic states also exhibits vibrational excitations. The basis representation of the dimer Hamiltonian is described in Appendix B.

### System-bath coupling Hamiltonian

From $N_{m}=N_{m,\mathrm{explicit}}+N_{m,\mathrm{bath}}$ vibrational modes in the monomer Hamiltonian in Eq. (1), $N_{m,\mathrm{explicit}}$ high-frequency modes are treated explicitly by including them in the system Hamiltonian, and the remaining $N_{m,\mathrm{bath}}$ low-frequency modes enter as contributions to a thermal bath in the framework of the concept of an open quantum system (May and Kühn 2011). The contribution of a bath mode with index *i* to the system-bath coupling Hamiltonian $\hat{H}_{\mathrm{SB}}$ is obtained by selecting the sum of the terms with linear dependence on $\hat{q}_{i}$ from expansion of the squared expression $(\hat{q}_{i}-d_{m,i})^2$, whereas the terms $\hat{q}_{i}^2$ and $d_{m,i}^2$ are attributed to the bath Hamiltonian $\hat{H}_{\mathrm{B}}$ and the system Hamiltonian $\hat{H}_{\mathrm{S}}$, respectively. In the bath Hamiltonian also the squared momentum operators $\hat{p}_{i}^2$ enter. The system Hamiltonian corresponds to the Hamiltonian of the dimer involving the explicitly treated modes, as specified above, with an energy correction of the basis states by the reorganization energies of the bath modes introduced by the term containing the squared displacement. The combination of the system-bath coupling contributions of the monomer units in a dimer system leads to

3 $$\begin{aligned} \hat{H}_{\mathrm{SB}}=\sum _{\bar{a} \in \{ 1,2 \}} \sum _{\bar{m} \in \{ \mathrm{S_1},\mathrm{S_2},\mathrm{S_3},\mathrm{S_4} \}} \sum _i^{N_{m,\mathrm{bath}}} \omega _{i}^{2} d_{\bar{m},i}^{(\bar{a})} \hat{q}_{i}^{(\bar{a})} \mid \bar{m}^{(\bar{a})} \rangle \langle \bar{m}^{(\bar{a})} \mid . \end{aligned}$$

The basis representation of this operator is formulated in Appendix C, where also the treatment of the dissipative dynamics is described.

### Calculation of linear optical spectra

For the calculation of linear absorption spectra we introduce the lower and upper triangular part of the vectorial transition dipole operator in the exciton basis, $\hat{\vec {\mu }}_{\mathrm{exc,+}}=\sum _{\alpha } \sum _{\xi } \vec {e}_{\xi } \mu _{\mathrm{exc},\xi , \alpha 0} \mid \alpha \rangle \langle 0 \mid$ and $\hat{\vec {\mu }}_{\mathrm{exc,-}}=\sum _{\alpha } \sum _{\xi } \vec {e}_{\xi } \mu _{\mathrm{exc},\xi , 0 \alpha } \mid 0 \rangle \langle \alpha \mid$, where $\vec {e}_{\xi }$ is the unit vector in direction $\xi$. We furthermore introduce a time evolution operator $\hat{U}(t)$, which comprises the evolution of coherent and dissipative dynamics and is evaluated by time propagation of the QME, in practice. Initially, all elements of the system density matrix are zero, apart from the ground state population. We denote the initial system density matrix as $\hat{\rho }_{0,g}$ and assume an initial equilibration of the thermal bath with density matrix $\hat{\rho }_{\mathrm{B,eq}}$. With these definitions we can write the dipole-dipole correlation function of absorption as

4 $$\begin{aligned} C_{\mathrm{abs}}(t)=\langle \langle \hat{\vec {\mu }}_{-} \hat{U}(t) \hat{\vec {\mu }}_{+} \hat{\rho }_{0,g} \rangle \rangle , \end{aligned}$$

where $\langle \langle \bullet \rangle \rangle =Tr_S \left\{ Tr_B \left\{ \bullet \hat{\rho }_{\mathrm{B,eq}} \right\} \right\}$ denotes the trace over both system and bath. As we take the transition dipole operators as vectorial quantities, the contributions of the directional components are separated from each other. Therefore, an orientational average is obtained by multiplication with a global factor of $\frac{1}{3}$. Furthermore, to take inhomogeneous broadening into account, we vary the electronic excitation energies of the monomer units (site energies) independent of each other by adding random numbers from a Gaussian distribution. This approach is equivalent to an independent incremental variation of the site energies and subsequent weighting of the resulting spectra by the respective Gaussian distribution function.

In the calculation of circular dichroism (CD) spectra not only the electronic transition dipole moments, which under the assumption of localized excitation of electronic state *n* of a single monomer unit *a* from the electronic ground state are given as $\vec {\mu }^{(a)}_{n 0}=\sum _{\xi} \mu ^{(a)}_{\xi ,n 0} \vec {e}_{\xi }$, but also the magnetic transition dipole moments enter. The latter are imaginary and proportional to the cross product of the position vector $\vec {R}_a$ of monomer unit *a*, where the position is identified with the center of mass, and the corresponding electronic transition dipole moment: $\vec {m}^{(a)}_{n 0} \sim i \vec {R}_a \times \vec {\mu }^{(a)}_{n 0}$ (Lindorfer and Renger 2018; Weigang 1965). Note that we neglect the small intrinsic CD of Chl *a* (Lindorfer et al. 2017). In analogy to the case of the electronic transition dipole moment an operator of the magnetic transition dipole moment in the basis of the electronic states for each directional component $\xi$ can be defined as $\hat{m}^{(\bar{a})}_{\xi }=\sum _{\bar{n}} m^{(\bar{a})}_{\xi ,\bar{n} 0} \mid \bar{n}^{(\bar{a})} \rangle \langle 0^{(\bar{a})} \mid + c.c$. Accordingly, after transformation to the exciton basis one obtains the lower and upper triangular part of the vectorial magnetic transition dipole operators $\hat{\vec {m}}_{\mathrm{exc,+}}=\sum _{\alpha } \sum _{\xi } \vec {e}_{\xi } m_{\mathrm{exc},\xi , \alpha 0} \mid \alpha \rangle \langle 0 \mid$ and $\hat{\vec {m}}_{\mathrm{exc,-}}=\sum _{\alpha } \sum _{\xi } \vec {e}_{\xi } m_{\mathrm{exc},\xi , 0 \alpha } \mid 0 \rangle \langle \alpha \mid$, respectively. In analogy to Eq. (4) we formulate the dipole-dipole correlation function of CD as

5 $$\begin{aligned} C_{\mathrm{CD}}(t)=\langle \langle \hat{\vec {m}}_{-} \hat{U}(t) \hat{\vec {\mu }}_{+} \hat{\rho }_{0,g} \rangle \rangle . \end{aligned}$$

The absorption and CD spectrum is obtained via Fourier transformation as

6 $$\begin{aligned} \sigma _{\mathrm{abs}}(\omega )=\Re \left\{ \int _0^{\infty } \mathrm{{d}}t \exp (i \omega t) C_{\mathrm{abs}}(t) \right\} , \end{aligned}$$

and

7 $$\begin{aligned} \sigma _{\mathrm{CD}}(\omega )=\Im \left\{ \int _0^{\infty } \mathrm{{d}}t \exp (i \omega t) C_{\mathrm{CD}}(t) \right\} , \end{aligned}$$

respectively.

The correlation functions $C_{\mathrm{abs}}(t)$ and $C_{\mathrm{CD}}(t)$ are obtained as (see Appendix 9)

8 $$\begin{aligned} C_{\mathrm{abs}}(t)=\sum _{\alpha } \sum _{\xi } \mu _{\mathrm{exc},\xi , 0 \alpha } C_{\alpha }(t), \end{aligned}$$

and

9 $$\begin{aligned} C_{\mathrm{CD}}(t) \sim \sum _{\alpha } \sum _{\xi } m_{\mathrm{exc},\xi , 0 \alpha } C_{\alpha }(t) \end{aligned}$$

with

10 $$\begin{aligned} \begin{aligned}&C_{\alpha }(t)= \mu _{\mathrm{exc},\xi , \alpha 0} \exp (-i \tilde{\omega }_{\alpha 0} t) \exp (G_{\alpha \alpha }(t)-G_{\alpha \alpha }(0)) \exp (-t/\tau _{\alpha }), \end{aligned} \end{aligned}$$

which contains the renormalized excitation energy of exciton state $\mid \alpha \rangle$,

11 $$\begin{aligned} \tilde{\omega }_{\alpha 0}=\omega _{\alpha 0} -\gamma _{\alpha \alpha \alpha \alpha } \lambda +\sum _{\beta \ne \alpha } \gamma _{\alpha \beta \beta \alpha } \Im (\tilde{C}(\omega _{\alpha \beta })) \end{aligned}$$

and

12 $$\begin{aligned} \tau _{\alpha }=\Big [ \frac{1}{2} \sum _{\beta } \Gamma _{\mathrm{ltb},\alpha \beta } \Big ]^{-1} \end{aligned}$$

with

13 $$\begin{aligned} \Gamma _{\mathrm{ltb},\alpha \beta }=2 \gamma _{\alpha \beta \beta \alpha } \Re (\tilde{C}(\omega _{\alpha \beta })). \end{aligned}$$

Here, $\gamma _{\alpha \beta \beta \alpha }$ is given as $\sum _m \sum _n \hat{A}_{\alpha m} \hat{A}_{m \beta } \hat{A}_{\beta n} \hat{A}_{n \alpha }$ and thus corresponds to products of coefficients entering in the transformation from the localized basis to the exciton basis, $\tilde{C}(\omega _{\alpha \beta })$ is the Fourier transform of the system-bath correlation function introduced in Appendix C and $\lambda$ is the reorganization energy of a local optical excitation.

## Model assumptions and parameters

The model system for the monomer which we use for our calculations is adopted from Reimers et al. (2013). However, we apply some modifications, such as a reduction of the number of intramolecular vibrational modes attributed to the system (see Table 1) and additional involvement of modes attributed to a thermal bath. Furthermore, we extend the model in such a way that it is also capable of describing an excitonic dimer in the framework of the OPA. As we are aiming at a validation of the model by comparison with measured spectra of Chl *a* from the literature, we adopt the model parameters of the monomer specified in Reimers et al. (2013) for Chl *a* dissolved in ether. Accordingly, we assume an energy gap between electronic excitation of Q$_{\mathrm{x}}$ and Q$_{\mathrm{y}}$ as $\Delta E_{\mathrm{Q_x} \mathrm{Q_y}}=\epsilon _{\mathrm{Q_x}}-\epsilon _{\mathrm{Q_y}}=1640\,{\hbox {cm}^{-1}}$, a ratio of the squared optical transition dipole moments for electronic excitation of Q$_{\mathrm{x}}$ and Q$_{\mathrm{y}}$ as $f_{\mathrm{Q_x} \mathrm{Q_y}}=0.1$ and the full width at half maximum (FWHM) for the inhomogeneous broadening of Q$_{\mathrm{x}}$ and Q$_{\mathrm{y}}$ as $\mathrm{FWHM}(\mathrm{Q_x})=720\,{\hbox {cm}^{-1}}$ and $\mathrm{FWHM}(\mathrm{Q_y})=360\,{\hbox {cm}^{-1}}$, respectively. To compensate the influence of the bath modes, which are not taken into account in Reimers et al. (2013), we take the liberty of adjusting the inhomogeneous broadening width of Q$_{\mathrm{y}}$ to $\mathrm{FWHM}(\mathrm{Q_y})=240\,{\hbox {cm}^{-1}}$. For the description of a dimer of Chl *a* bound to WSCP the inhomogeneous broadening is modified anyway, and values of $\mathrm{FWHM}(\mathrm{Q_x})=340\,{\hbox {cm}^{-1}}$ and $\mathrm{FWHM}(\mathrm{Q_y})=170\,{\hbox {cm}^{-1}}$ are assumed. The latter value has been determined previously (Renger et al. 2007), whereas for the former we assume that the relative magnitude with respect to the width of the Q$_{\mathrm{y}}$ transition remains the same as in the solvent described above. The $\mathrm{B_y} (\mathrm{S_0} \rightarrow \mathrm{S_3})$ and $\mathrm{B_x} (\mathrm{S_0} \rightarrow \mathrm{S_4})$ transitions, which are decoupled from Q$_{\mathrm{y}}$ and Q$_{\mathrm{x}}$ transitions of the same monomer, are energetically shifted with respect to Q$_{\mathrm{y}}$ by $\Delta E_{\mathrm{B_y} \mathrm{Q_y}}=7570\,{\hbox {cm}^{-1}}$ and $\Delta E_{\mathrm{B_x} \mathrm{Q_y}}=8740\,{\hbox {cm}^{-1}}$, and the ratios of the transition dipole strengths of B$_{\mathrm{y}}$ and B$_{\mathrm{x}}$ and of Q$_{\mathrm{y}}$ are $f_{\mathrm{B_y} \mathrm{Q_y}}=2.52$ and $f_{\mathrm{B_x} \mathrm{Q_y}}=2.43$, respectively, as determined previously (Lindorfer et al. 2017). Note, however, that the exact energies of the B_x_ and B_y_ states are not so critical, since we want to study the influence of these transitions on the low-energy (Q_y_ and Q_x_) region of the spectra. According to Reimers et al. (2013), we assume a vibronic coupling constant of $\tilde{\alpha }_{\mathrm{VC}}=750\,{\hbox {cm}^{-1}}$ and a frequency of the vibronic coupling mode of $\omega _{\mathrm{VC}}=1500\,{\hbox {cm}^{-1}}$. However, different from Reimers et al. (2013), we further reduce the number of intramolecular vibrational modes included in the model. Starting from the 51 effective vibrational modes identified there, we compose nine groups of five modes and one group with six modes by appropriately partitioning the modes sorted by their frequencies and replace each group by a single mode with summed Huang–Rhys factor and averaged frequency, where in the calculation of the frequency average a weighting by the Huang–Rhys factors of the respective modes is applied. The summed Huang–Rhys factors of the ten resulting modes are then attributed to the involvement of the respective modes in the Q$_{\mathrm{y}}$ transition. The frequencies and Huang–Rhys factors of the resulting modes are composed in Table 1.

**Table 1:** Explicitly treated intramolecular vibrational modes *i* with their respective frequencies $\omega _i$ and Huang–Rhys factors $S_i$. These modes were obtained by separation of sets of five (or six) modes with similar frequencies from the 51 modes given in Reimers et al. (2013), thereby calculating a summed Huang–Rhys factor and an averaged frequency, where in the calculation of the latter the frequency contribution of each mode in a selected set is weighted by the relative contribution to the Huang–Rhys factor of the combined mode. The sum of the Huang–Rhys factors is 0.278, as in Reimers et al. (2013)

i	1	2	3	4	5	6	7	8	9	10
$\omega _i[cm^{-1}]$	169	345	438	572	732	843	993	1197	1310	1511
$S_i*10^{3}$	29.5	22.2	4.4	10.3	27.2	18.2	47.4	29.9	52.1	36.7

In the case of the Q$_{\mathrm{x}}$ transition the Huang–Rhys factors of the lowest-frequency modes ($i=1,2,3$) are rescaled in such a way that the displacement of these modes in the Q$_{\mathrm{x}}$ transition is three times larger than in the Q$_{\mathrm{y}}$ transition, as suggested in Reimers et al. (2013), leading to a factor of 9 for the Huang–Rhys factors. This rescaling in the low-frequency range leads to an increase of the total reorganization energy from $262\,{\hbox {cm}^{-1}}$ for Q$_{\mathrm{y}}$ to $380\,{\hbox {cm}^{-1}}$ for Q$_{\mathrm{x}}$. The system-bath coupling is determined by the spectral density given in Eq. (C18), where in comparison with Renger and Marcus (2002) the Huang–Rhys factors are rescaled in such a way that the total Huang–Rhys factor becomes equal to 0.8, as determined previously from the temperature dependence of the absorption spectrum of WSCP (Renger et al. 2007). This rescaling leads to the values $s_1=0.402$ and $s_2=0.398$, while the corresponding frequencies $\omega _1=0.557\,{\hbox {cm}^{-1}}$ and $\omega _2=1.94\,{\hbox {cm}^{-1}}$ remain unchanged. To determine the directions of the transition dipole moments we rely on structural data for the WSCP (Horigome et al. 2007) and determine the vectors connecting the nitrogen atoms at opposite sites of the Chl *a* molecules. While the transition dipole moment $\vec {\mu }_{\mathrm{Q_y}}$ is supposed to be aligned with the connecting vector of the nitrogen atoms identified as $\mathrm{N_B}$ and $\mathrm{N_D}$, the transition dipole moment $\vec {\mu }_{\mathrm{Q_x}}$ is associated with the connecting vector of the nitrogen atoms identified as $\mathrm{N_A}$ and $\mathrm{N_C}$. In fact, this rule of thumb merely gives some orientation. In previous works it turned out that a more accurate description is obtained by applying a rotation of the transition dipole moment in the plane spanned by the connecting vectors of the nitrogen atoms. More precisely, in the case of $\vec {\mu }_{\mathrm{Q_y}}$ a rotation by $-7{^{\circ }}$ has turned out to be appropriate to reproduce the measured rotational strength of the dimer in WSCP by calculations (Renger et al. 2009). In the present work also rotations of $\vec {\mu }_{\mathrm{Q_x}}$, $\vec {\mu }_{\mathrm{B_y}}$ and $\vec {\mu }_{\mathrm{B_x}}$ by $20{^{\circ }}$, $20{^{\circ }}$ and $-20{^{\circ }}$ have been applied, respectively, as these values have turned out to be the most appropriate ones for simulation of the measured spectra (see Supporting Information (SI), Section III, Figs. S9–S16). Note, however, that these rotations are not critical for the optical spectra. Qualitatively similar spectra are obtained if $\vec {\mu }_{\mathrm{Q_x}}$, $\vec {\mu }_{\mathrm{B_y}}$ and $\vec {\mu }_{\mathrm{B_x}}$ are assumed to be oriented along the *x*-, *y*-, and *x*-axes, respectively (SI, Fig. S17). For the excitonic couplings between the electronic transitions of the different monomer units we used the following estimates: For the coupling between the Q$_{\mathrm{y}}$ 0-0 transitions we use $J_{\mathrm{Q_y} \mathrm{Q_y}}=83\,{\hbox {cm}^{-1}}$. This value has been obtained from a fit of optical spectra (Adolphs et al. 2016) and, recently from quantum chemical/electrostatic calculations (Friedl et al. 2022). In dipole-dipole approximation the excitonic coupling is given as

14 $$\begin{aligned} J_{mn}=\frac{\mu ^{(a)}_{m} \mu ^{(b)}_{n}}{R_{ab}^3} \zeta _{mn}^{(a,b)}, \end{aligned}$$

where $R_{ab}=\mid \vec {R}^{(b)}-\vec {R}^{(a)} \mid$ is the center-to-center distance between monomers *a* and *b* and the additional factor $\zeta _{mn}^{(a,b)}$ reads

15 $$\begin{aligned} \zeta _{mn}^{(a,b)}=\vec {e}_m^{(a)} \cdot \vec {e}_n^{(b)} -3 (\vec {e}_m^{(a)} \cdot \vec {e}_{ab}) (\vec {e}_n^{(b)} \cdot \vec {e}_{ab}). \end{aligned}$$

Here, $\vec {e}_m^{(a)}$, $\vec {e}_n^{(b)}$ and $\vec {e}_{ab}$ are vectors along $\vec {\mu }_m^{(a)}$, $\vec {\mu }_n^{(b)}$ and $\vec {R}^{(b)}-\vec {R}^{(a)}$, respectively. For the present system, the dipole-dipole approximation gives a deviation of about $10{\%}$ for the coupling between the $\mathrm{Q_y}$ transitions $J_{\mathrm{Q_y} \mathrm{Q_y}}$ (Friedl et al. 2022). This deviation is small enough to estimate the excitonic couplings between the remaining transitions as

16 $$\begin{aligned} J_{mn}=J_{\mathrm{Q_y} \mathrm{Q_y}} f_{m, \mathrm{Q_y}}^{(a)} f_{n, \mathrm{Q_y}}^{(b)} \frac{\zeta _{mn}^{(a,b)}}{\zeta _{\mathrm{Q_y} \mathrm{Q_y}}^{(a,b)}}, \end{aligned}$$

where $f_{k, \mathrm{Q_y}}^{(c)}=\frac{\mu ^{(c)}_{k}}{\mu ^{(c)}_{\mathrm{Q_y}}}$ is the ratio of transition dipole magnitudes between the *k*-th and the $\mathrm{Q_y}$ transition of monomer *c*. The resulting values of the excitonic coupling in the subspace of Q$_{\mathrm{x}}$ and Q$_{\mathrm{y}}$ excitation are $J_{\mathrm{Q_x} \mathrm{Q_x}}=-2.8\,{\hbox {cm}^{-1}}$ and $J_{\mathrm{Q_x} \mathrm{Q_y}}=4.9\,{\hbox {cm}^{-1}}$. Furthermore, the values $J_{\mathrm{B_x} \mathrm{B_x}}=-24.8\,{\hbox {cm}^{-1}}$, $J_{\mathrm{B_x} \mathrm{B_y}}=-73.9\,{\hbox {cm}^{-1}}$, $J_{\mathrm{B_y} \mathrm{B_y}}=205.2\,{\hbox {cm}^{-1}}$, $J_{\mathrm{B_x} \mathrm{Q_x}}=-19.9\,{\hbox {cm}^{-1}}$, $J_{\mathrm{B_x} \mathrm{Q_y}}=-68.5\,{\hbox {cm}^{-1}}$, $J_{\mathrm{Q_x} \mathrm{B_y}}=15.5\,{\hbox {cm}^{-1}}$ and $J_{\mathrm{Q_y} \mathrm{B_y}}=136.2\,{\hbox {cm}^{-1}}$ are obtained. If we are aiming at a comparison of calculated spectra with measured ones, we adjust the energetic position of Q$_{\mathrm{y}}$ accordingly, otherwise we set it to zero.

As mentioned in  Section  “Monomer Hamiltonian” we only take a very limited number of vibrational excitations into account. While only a single intramolecular vibration can be excited to its first vibrational eigenstate, excitation of up to four vibrational quanta of the vibronic coupling mode is possible in the framework of our treatment. The applied restriction with respect to the number of intramolecular vibrational excitations can be justified by the small Huang–Rhys factors of the respective modes. Even though there is no displacement of the vibronic coupling mode upon electronic excitation, the vibronic coupling leads to an effective displacement in the diagonalized Hamiltonian. As the corresponding Huang–Rhys factor is estimated to be larger than the Huang–Rhys factors of the individual intramolecular vibrational modes, we take four excited vibrational eigenstates into account to ensure convergence. Please note that simultaneous excitation of an intramolecular vibrational mode and of the vibronic coupling mode is feasible in our treatment. Such combined excitations gain relevance in the side band region of absorption and CD spectra. However, including them drastically increases the numerical effort, making convergence tests with respect to the number of vibrational modes, included in the model, and with respect to restrictions regarding the number of vibrationally excited basis states difficult. We therefore applied such tests only at an earlier stage of the development of our model, where simultaneous excitation of an intramolecular vibrational mode and of the vibronic coupling mode had not been included yet. Some of the respective results are shown in Section IB of the SI.

It is worth mentioning that the vibronic coupling relies on the concept of a conical intersection. For the description of conical intersections separable tuning and a coupling modes can be identified (Robb 2011), where only the latter actually couples the involved electronic states and thus corresponds to the vibronic coupling mode in our description. For a tuning mode different Huang–Rhys factors in the involved electronic states are required. This criterion is fulfilled for the first three modes with the lowest frequencies from the ten recombined modes in our description. As we treat the vibronic coupling mode independent of the specific vibrational modes with the character of tuning modes (and also independent of the remaining vibrational modes), thereby accounting for simultaneous excitations of these different types of modes in the product basis of the vibrational eigenstates, our description is compatible with the concept of a conical intersection which implies the independence of coupling and tuning mode.

## Results and discussion

To illustrate the influence of different aspects of the model on the optical spectra of Chl *a* monomers in ether (Fig. 1) and of Chl *a* dimers in WSCP (Fig. 2), we start with a minimal model and successively extend it until all relevant aspects are included.

### Monomer absorption spectra

**Fig. 3:**
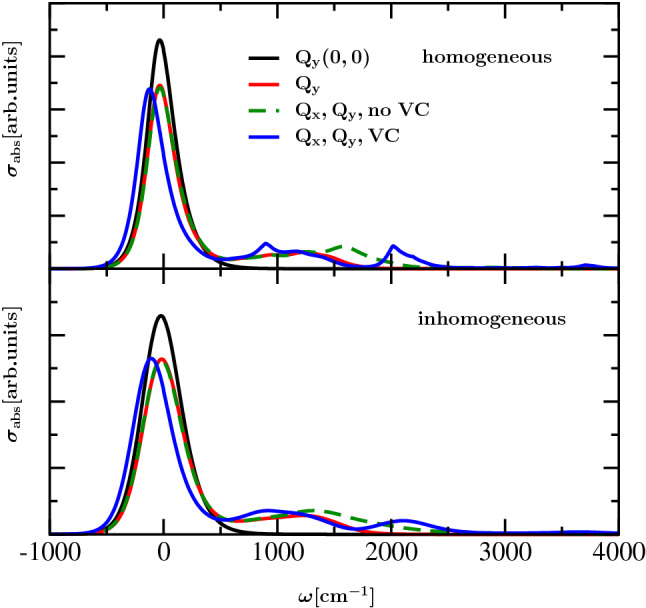
Absorption spectra of Chl *a* monomer at $T=300\,{\hbox {K}}$ without and with inhomogeneous broadening are displayed in upper and lower panel, respectively; black line: without intramolecular vibrations, excitation of Q$_{\mathrm{y}}$ only; red line: with intramolecular vibrations, excitation of Q$_{\mathrm{y}}$ only; green line: with intramolecular vibrations, excitation of Q$_{\mathrm{y}}$ and Q$_{\mathrm{x}}$; blue line: with additional vibronic coupling between the Q$_{\mathrm{x}}$ and Q$_{\mathrm{y}}$ transitions

The homogeneous and inhomogeneous absorption spectra of the Chl *a* monomer resulting from various approximations are displayed in the upper and lower panel of Fig. 3, respectively. First we consider a very simplified situation, where both the vibronic coupling mode and the intramolecular vibrational modes are disregarded by setting the vibronic coupling constant and the assigned Huang–Rhys factors to zero, respectively, so that the influence of vibrational dynamics only enters in terms of components associated with the thermal bath. Under the assumption that only Q$_{\mathrm{y}}$ is excited without involvement of its vibrational structure, only a single peak appears in the absorption spectrum displayed as a black line. This peak is centered at zero on the frequency axis, which corresponds to the electronic excitation energy of the Q$_{\mathrm{y}}$ transition. If the coupling of the intramolecular vibrations to the Q$_{\mathrm{y}}$ excitation is taken into account by assuming the Huang–Rhys factors in Table 1, the peak centered at zero, which stems from the 0-0 transition, decreases, and due to the involvement of vibrational excitation of the different intramolecular modes a side band appears, as indicated by the red line. Additional involvement of the Q$_{\mathrm{x}}$ transition with its vibrational structure leads to an enhancement of the absorption profile in the energetic region of Q$_{\mathrm{x}}$ (green and red line). Furthermore, if vibronic coupling between the Q$_{\mathrm{x}}$ and Q$_{\mathrm{y}}$ transitions is taken into account, the resulting absorption spectrum, displayed as a blue line, exhibits a slightly redshifted 0-0 line and substantial changes in the vibrational structure. Due to the vibronic coupling a band splitting occurs with new bands around $1000\,{\hbox {cm}^{-1}}$ and $2000\,{\hbox {cm}^{-1}}$. The splitting appears because of the coupling between the first excited vibrational eigenstate of the vibronic coupling mode in S$_1$ and the lowest vibrational eigenstate of the vibronic coupling mode in S$_2$, which are energetically close to each other and give rise to a quantum mechanical mixing between the 0-1 Q$_{\mathrm{y}}$ transition and the 0-0 Q$_{\mathrm{x}}$ transition. The origin of the resulting peaks in the monomer absorption spectrum is discussed Section IA in the SI. As we take simultaneous excitation of the vibronic coupling mode and of a single intramolecular mode to its first vibrational eigenstate into account, also the intramolecular modes get involved the vibronic coupling, even though the influence of simultaneous excitation turns out to be negligible from a comparison of Figs. S1 and S2 from the SI. A minor contribution to the peak in the region above $2000\,{\hbox {cm}^{-1}}$ and the small peak above $3500\,{\hbox {cm}^{-1}}$ can be associated with the Q$_{\mathrm{y}}$ transition under the influence of vibronic coupling.

If inhomogeneous broadening is involved, the absorption spectra become smoother, and the peaks are spread over a broader frequency range, as displayed in the lower panel of Fig. 3. From the comparison of the measured absorption spectrum for Chl *a* in ether Reimers et al. (2013) and the calculated result in Fig. 1 (involving adjustment of the energetic position of the zero phonon line to the actual transition frequency of Q$_{\mathrm{y}}$ excitation) it becomes recognizable that the involvement of vibronic coupling is required to reproduce the progression of the side bands. Note that the involvement of all 51 modes from Reimers et al. (2013) instead of the ten recombined modes did not lead to recognizable changes in the absorption spectra (see Section IB and Fig. S3 in the SI). In Section IB of the SI we also discussed whether taking into account only the first excited state of a single intramolecular vibrational mode is sufficient. The excitation of more than one vibrational quantum does not lead to qualitative changes in the shape of the spectra (SI, Section IB).

### Dimer absorption spectra

**Fig. 4:**
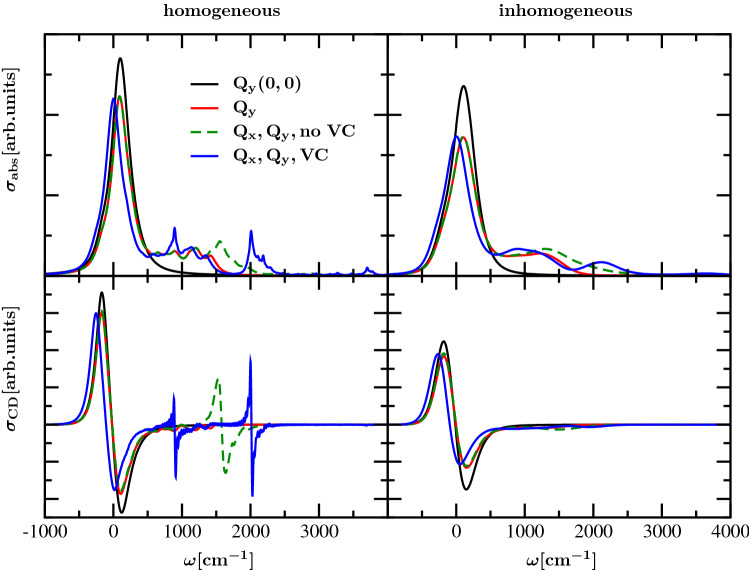
Absorption (upper panels) and CD spectra (lower panels) of dimer at $T=300\,{\hbox {K}}$ without and with inhomogeneous broadening (left and right panels, respectively); black line: without any vibrations, excitation of Q$_{\mathrm{y}}$ only; red line: with intramolecular vibrations, excitation of Q$_{\mathrm{y}}$ only; green line: with intramolecular vibrations, excitation of Q$_{\mathrm{y}}$ and Q$_{\mathrm{x}}$; blue line: with additional vibronic coupling between Q$_{\mathrm{x}}$ and Q$_{\mathrm{y}}$ transitions

Next we study the properties of the dimer model with excitonic coupling between the monomer units, aiming at an understanding of the measured (Palm et al. 2017) and calculated spectra in Fig. 2. We disregard inhomogeneous broadening at first and successively take into account the involvement of different aspects of the model in analogy to the corresponding discussion in the monomer case (Fig. 3). The resulting homogeneous absorption and circular dichroism spectra are shown in the upper and lower left half of Fig. 4, respectively. The corresponding inhomogeneous spectra are shown in the right half of Fig. 4. It turns out that the changes of the dimer absorption spectrum in the region of intramolecular vibrational sidebands are similar to those of the monomer for the considered cases. This finding can be explained by the relatively small values of the excitonic couplings compared to the frequencies of the vibronic coupling mode and of the explicitly treated intramolecular vibrations. Recognizable differences between the absorption spectra of dimer and monomer consist in the less smooth vibrational side bands in the dimer case due to underlying splitting of the excitonically coupled states with a single intramolecular vibrational excitation. The relative amplitude of the peak in the 0-0 Q$_{\mathrm{y}}$ region with respect to that of the high-frequency vibrational sidebands (and the Q$_{\mathrm{x}}$ transition) is smaller in the case of the dimer (Figs. 2 and 4) than in the case of the monomer (Figs. 1 and 3). This effect is caused by the excitonic splitting between the exciton states formed by the 0-0 Q$_{\mathrm{y}}$ transitions and the strong lifetime broadening of the upper exciton state (Renger et al. 2007). Obviously, the lifetime broadening of the (mixed Q$_{\mathrm{y}}$–Q$_{\mathrm{x}}$) excited states is smaller.

In all of the considered cases in the homogeneous CD spectrum (lower left panel of Fig. 4) a pair of opposite-signed peaks appears close to the zero position associated with the electronic excitation energy of the 0-0 Q$_{\mathrm{y}}$ transitions. In the green curve, which is obtained under the assumption of involvement of Q$_{\mathrm{x}}$ excitation with its vibrational structure, a peak combination with a sign change appears at the electronic excitation energy of Q$_{\mathrm{x}}$ (around $1500\,{\hbox {cm}^{-1}}$). These peak structures in the energetic region of the Q$_{\mathrm{y}}$ and Q$_{\mathrm{x}}$ transitions can be attributed to the influence of the excitonic couplings $J_{\mathrm{Q_y} \mathrm{Q_y}}$ and $J_{\mathrm{Q_x} \mathrm{Q_x}}$, respectively. From the point of view that $J_{\mathrm{Q_x} \mathrm{Q_x}}$ is more than a magnitude smaller than $J_{\mathrm{Q_y} \mathrm{Q_y}}$ it seems surprising that the integral over the absolute value of the peak structures in the energetic region of Q$_{\mathrm{x}}$ still corresponds to about $\frac{1}{3}$ of the integral over the peak structures in the energetic region of the 0-0 Q$_{\mathrm{y}}$ transitions. Obviously, the influence of the arrangement of the transition dipole moments involved in the respective transitions overcompensates the tendencies expected for the relative peak intensities by relying on the absolute values of the excitonic coupling matrix elements. Switching on the vibronic coupling between the Q$_{\mathrm{y}}$ and Q$_{\mathrm{x}}$ transitions leads to a slight redshift of the CD peaks in the Q$_{\mathrm{y}}(0,0)$ region and to a splitting of the Q$_{\mathrm{x}}(0,0)$ CD peaks with new peaks at around $1000\,{\hbox {cm}^{-1}}$ and $2000\,{\hbox {cm}^{-1}}$. The rotational strength in the vicinity of the two new peaks is reduced with respect to that of the Q$_{\mathrm{x}}(0,0)$ transition observed in the absence of vibronic coupling. Because of the mixing of Q$_{\mathrm{x}}$ and Q$_{\mathrm{y}}$ the aspect of the different geometric arrangement of the assigned transition dipole moments gains importance, resulting in a decrease of the integrated CD spectrum due to the involvement of Q$_{\mathrm{y}}$ by its vibronic coupling to Q$_{\mathrm{x}}$.

By including inhomogeneous broadening the spectra become considerably smoother (right hand side of Fig. 4). It becomes recognizable that in the energetic regions where side bands with considerable intensity appear in the absorption spectra, the relative intensity with respect to the spectral region of the 0-0 Q$_{\mathrm{y}}$ transitions is much lower in the CD spectra. Obviously the sign changes in the peaks, which were observed in the CD spectrum without inhomogeneous broadening, result in cancelation when inhomogeneous broadening gets involved. In addition, the site energy disorder leads to a localization of the exciton states involving excited intramolecular vibrations, because of their small Franck-Condon factors that give rise to a small excitonic coupling. These localized excited states contribute only to the absorption, but not to the circular dichroism spectrum.

**Fig. 5:**
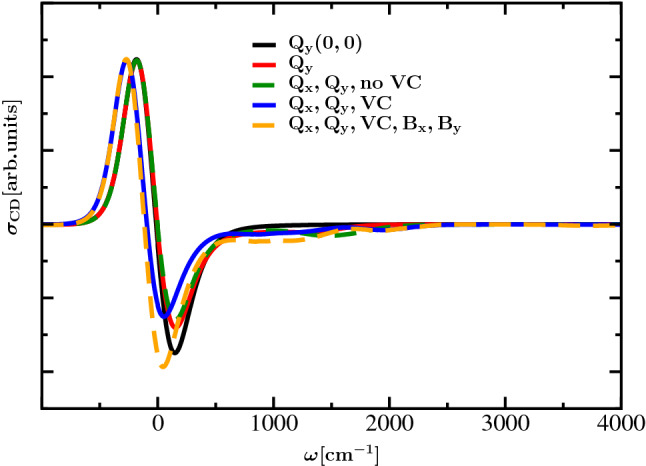
CD spectra of dimer at $T=300\,{\hbox {K}}$ from the lower right panel of Fig. 4 are rescaled in such a way that the positive-signed peak has the same amplitude. Different from Fig. 4, also a case with additional involvement of $\mathrm{B_x}$ and $\mathrm{B_y}$ is displayed as an orange dashed line

Interestingly, the coupling of the Q$_{\mathrm{y}}$ transition to intramolecular modes leads to a non-conservative shape of the CD spectrum in the 0-0 spectral region of the Q$_{\mathrm{y}}$ transition, which means that integration over the selected spectral region does not lead to compensation of contributions from positive- and negative-signed peaks (Lindorfer et al. 2017; Georgakopoulou et al. 2002, 2006, 2007; Lindorfer et al. 2021). This property becomes recognizable from the colored lines in the lower right panel of Fig. 4. There is redistribution of negative rotational strength of the 0-0 transition to the high-frequency vibrational sideband region that is hardly visible in Fig. 4 because of the large spectral width of the sideband region. Since the experimental CD spectrum of Chl *a* WSCP is conservative in the 0-0 Q$_{\mathrm{y}}$ spectral region (Fig. 2), an additional mechanism seems to be active in the experiment. Such a mechanism is provided by the coupling of Q$_{\mathrm{x}}$ and Q$_{\mathrm{y}}$ to B$_{\mathrm{x}}$ and B$_{\mathrm{y}}$. In order to quantify this effect, we have rescaled all inhomogeneous CD spectra from the lower right panel of Fig. 4, such that their positive peaks get equal amplitude. The resulting spectra are compared in Fig. 5 with a calculation that includes the excitonic couplings to high-energy B$_{\mathrm{x}}$ and B$_{\mathrm{y}}$ transitions (the orange line in Fig. 5). While the involvement of B$_{\mathrm{x}}$ and B$_{\mathrm{y}}$ plays a minor role in the absorption spectra (SI, Fig. S4, upper half), it has a substantial influence on the intensities of the positive- and negative-signed peak component in the energetic region of Q$_{\mathrm{y}}$ in the CD spectrum (SI, Fig. S4, lower half). As a measure of the conservativity of the CD spectrum in the low-energy region we can take the spectrum resulting by including only the 0-0 transitions of the monomers (the black solid line in Fig. 5). Including the intramolecular vibrations of the Q$_{\mathrm{y}}$ transition (red line) leads to a redistribution of negative rotational strength from the main peak around 0 to the vibrational sideband around $1000\,{\hbox {cm}^{-1}}$. This redistribution persists upon including the Q$_{\mathrm{x}}$ transition and the vibronic coupling between Q$_{\mathrm{x}}$ and Q$_{\mathrm{y}}$. Including the B$_{\mathrm{x}}$ and B$_{\mathrm{y}}$ transitions, the main CD peaks finally get conservative again, in agreement with the experimental data in Fig. 2. In the SI the corresponding absorption and CD spectra are displayed over a wider frequency range which also captures the features resulting from excitation of B$_{\mathrm{x}}$ and B$_{\mathrm{y}}$ (SI, Fig. S4). Here we concentrate on the low-energy region.

**Fig. 6:**
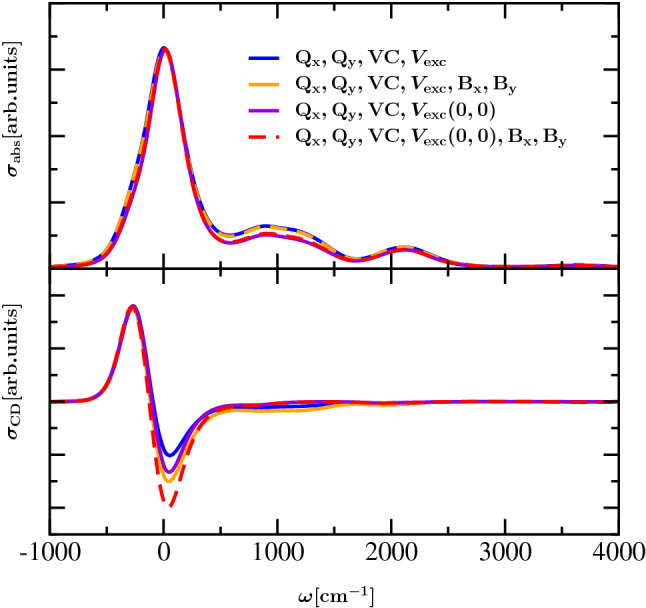
Absorption and CD spectra of dimer at $T=300\,{\hbox {K}}$ with inhomogeneous broadening ($\mathrm{FWHM}(\mathrm{Q_y})=240\,{\hbox {cm}^{-1}}$, $\mathrm{FWHM}(\mathrm{Q_x})=720\,{\hbox {cm}^{-1}}$), excitation of Q$_{\mathrm{y}}$ and Q$_{\mathrm{x}}$ and vibronic coupling are displayed in upper and lower panel, respectively; if all matrix elements of the excitonic coupling are taken into account, the blue and the orange line are obtained from calculations without and with involvement of B$_{\mathrm{y}}$ and B$_{\mathrm{x}}$ ($\mathrm{FWHM}(\mathrm{B_y})=\mathrm{FWHM}(\mathrm{B_x})=1800\,{\hbox {cm}^{-1}}$), respectively; if only those coupling matrix elements with involvement of lowest vibrational eigenfunctions are taken into account, the violet and the red line are obtained from calculations without and with involvement of B$_{\mathrm{y}}$ and B$_{\mathrm{x}}$, respectively. Please note that the absorption and CD spectra were rescaled to obtain a common maximum of the 0-0 line (the low-energy peak).

To get further insight into this mechanism which determines the appearance of the dimer spectra, we investigate the role of excitonic coupling involving vibrational excitation. To study the role of vibrational excitation in excitonic coupling, we include the intramolecular vibrational sidebands of the monomers, but include only the excitonic coupling between the 0-0 transitions of the monomers. The resulting CD spectra are composed in Fig. 6 together with the results of calculations that include all excitonic couplings which have already been discussed in the context of Figs. 4 and 5. The Q$_{\mathrm{x}}$ transition and its vibronic coupling to Q$_{\mathrm{y}}$ is included in all calculations and we investigate the involvement of the B$_{\mathrm{x}}$ and B$_{\mathrm{y}}$ transitions. In the absorption spectrum (upper part of Fig. 6) we find a slight decrease of the vibrational sideband around $1000\,{\hbox {cm}^{-1}}$ when excitonic coupling involving the 0-1 Q$_{\mathrm{y}}$ transitions is disregarded. Otherwise the vibrational sideband stays practically the same, independent of excitonic coupling to the high-energy B$_{\mathrm{x}}$ and B$_{\mathrm{y}}$ transitions. The small changes arising from involvement of the 0-1 Q$_{\mathrm{y}}$ transitions in the excitonic coupling indicate that the vibrational sideband is that of a monomer (including the vibronic mixing with the Q$_{\mathrm{x}}$ transition). This result is in agreement with a recent numerical study by Reppert (2020) on the character of excited states of molecular dimers and with a recent comparison of perturbative and numerically exact lineshape theories (Caycedo-Soler et al. 2022). It supports our earlier treatments (Renger et al. 2011; Müh and Renger 2012), where intramolecular excitations that include excitonic couplings as well as high-frequency vibrational excitations of pigment-protein complexes were treated as localized transitions, in contrast to alternative treatments that include high-frequency intramolecular modes into the spectral density and use perturbative line-shape theory that cannot distinguish between the different degrees of delocalization of the 0-0 and the 0-1 transitions of the chromophores (Gelzinis et al. 2017; Novoderezhkin et al. 2005).

The lower part in Fig. 6 contains the corresponding CD spectra (As in Fig. 5, for better comparison these spectra were rescaled to yield the same height of their positive peak). The results from calculations with and without contributions of vibrationally excited states in the excitonic coupling, both of them involving B$_{\mathrm{x}}$ and B$_{\mathrm{y}}$, are displayed with orange and red line color. Furthermore, the corresponding results for the case without involvement of B$_{\mathrm{x}}$ and B$_{\mathrm{y}}$ are displayed as a blue and a violet curve, respectively. As described above, there is redistribution of negative rotational strength between the 0-0 Q$_{\mathrm{y}}$ transition and the intramolecular vibrational side bands by the excitonic coupling between the 0-0 Q_y_ transition of one monomer and the 0-1 Q_y_ transition of the other monomer, where the increase of relative intensity of the side band is larger than in the absorption spectrum. Similar magnitudes of the negative peak in the CD spectrum are obtained from a full calculation (the orange line) and from a calculation where the coupling between the 0-0 Q$_{\mathrm{y}}$ transitions of one monomer and the B$_{\mathrm{x}}$ and B$_{\mathrm{y}}$ transitions as well as the 0-1 Q$_{\mathrm{y}}$ transitions of the other monomer are neglected (violet line). The coupling to B$_{\mathrm{x}}$ and B$_{\mathrm{y}}$ leads to a more negative rotational strength in the Q$_{\mathrm{y}}$ 0-0 region and the coupling to Q$_{\mathrm{y}}$ 0-1 shows opposite behavior. Hence, an error compensation occurs if both types of couplings are neglected.

Now that we have studied the influence of the different parts of our model Hamiltonian on the optical spectra of the Chl *a* dimer in WSCP, we are ready to discuss the comparison of our calculated spectra with the experimental data in Fig. 2 in detail. The experimental absorption spectrum shows a main peak at around $15000\,{\hbox {cm}^{-1}}$ and two broad peaks at $16000\,{\hbox {cm}^{-1}}$ and $17000\,{\hbox {cm}^{-1}}$. Our calculations show that the main peak is formed by two exciton transitions with dominant contributions from the 0-0 Q$_{\mathrm{y}}$ transitions of the two monomers. Due to the large homogeneous and inhomogeneous broadening these transitions appear as one peak at $300\,{\hbox {K}}$. At cryogenic temperatures the lowest exciton transition results in a low-energy shoulder of the main peak (see SI, Fig. S5). As in the monomer spectrum, the two broad high-energy peaks are mixed Q$_{\mathrm{y}}(0,1)$–Q$_{\mathrm{x}}(0,0)$ transitions. The excitonic coupling between the Q$_{\mathrm{y}}(0,1)$ transitions as well as between the Q$_{\mathrm{x}}$ transitions (and those between Q$_{\mathrm{y}}(0,1)$ and Q$_{\mathrm{x}}$) of the two monomers is found to have a negligible effect on the intensity and shape of the absorption spectrum. Overall, the agreement between theory and experiment is somewhat better for the absorption spectrum of the monomer (Fig. 1) than for that of the dimer (Fig. 2). This result is not surprising since the parameters of the vibronic coupling Hamiltonian of the monomer were fitted to the experiment (here Chl *a* in ether) by Reimers et al. (2013). We used these parameters also for Chl *a* in WSCP. The overall Huang–Rhys factor $S=\sum _i S_i=0.278$ (Table 1) is indeed close to the $S=0.23$ estimated recently (Friedl et al. 2022) from fluorescence line narrowing data of WSCP (Pieper et al. 2011a), taking into account the effect of the excitonic coupling on the redistribution of oscillator strength. Concerning the value of the vibronic coupling strength we do not have any independent estimate for Chl *a* in WSCP, but note that the values estimated by Reimers *et al.* for Chl *a* can vary by roughly $100{\%}$ depending on the solvent (see Table S9 from Reimers et al. (2013)). In the SI we analyze the dependence of the spectra on the variations of Huang–Rhys factors and vibronic coupling constants (SI Section II, Figs. S6–S8). The analysis suggests that reducing the vibronic coupling and increasing the Huang–Rhys factors somewhat improves the fit of the experiment. Our dimer spectra were calculated in the framework of the OPA, as discussed above. To explain the remaining differences between measured and calculated dimer spectra an extension of the model by also including vibrations in the electronic ground state of the de-excited monomer unit in the framework of the so-called two-particle approximation (TPA) (Spano 2002, 2010; Hestand and Spano 2018) could be revealing. Additional vibrational levels in the electronic ground state facilitate additional resonances between electronic de-excitation and excitation involving the vibrational substructure of excited states and ground state, which contribute to the excitonic coupling and would be expected to particularly influence the side band region. However, such an extension of the model would substantially increase the computational effort and would go beyond the scope of the present study. Preliminary calculations with application of the TPA only with respect to the vibronic coupling mode and without the possibility of simultaneous excitation of an intramolecular vibrational mode did not result in substantial differences compared to calculations with OPA, as discussed in Section IB of the SI.

Excellent agreement between theory and experiment is obtained for the CD spectrum (Fig. 2, lower part). In particular, the calculations explain the conservative nature of the spectrum and the absent signal in the high-energy spectral region. Our calculations show that the conservative nature of the CD spectrum rests on a delicate balance between different excitonic couplings between the Q$_{\mathrm{y}}(0,0)$ transition and higher electronic (B$_{\mathrm{x}}$, B$_{\mathrm{y}}$) and vibronic (Q$_{\mathrm{y}}(0,1)$–Q$_{\mathrm{x}}(0,0)$) transitions. In earlier studies the focus has been on the coupling of the Q$_{\mathrm{y}}(0,0)$ transition of Chl and bacteriochlorophyll (BChl) with higher electronic states of Chls, BChls and carotenoids (Lindorfer et al. 2017; Georgakopoulou et al. 2002, 2006, 2007). Since these transitions have a much larger dipole strength than the Q$_{\mathrm{y}}(0,1)$ or the Q$_{\mathrm{x}}(0,0)$ transition, it could be expected that their influence is larger. However, the present study shows that at least for Chl *a* these low-intensity transitions can compensate their small dipole strength by a much smaller energy difference with respect to the Q$_{\mathrm{y}}(0,0)$ transition. Often in the earlier studies only part of the non-conservativity could be explained and the missing part was assumed to originate from the intrinsic circular dichroism of the pigments. It could well be that for different dipole geometries the coupling to high-energy electronic transitions and that to Q$_{\mathrm{y}}(0,1)$ and Q$_{\mathrm{x}}(0,0)$ give non-conservative contributions in the Q$_{\mathrm{y}}(0,0)$ region that add up instead of compensating each other as in the present study. The present calculations also explain why there is practically no CD signal outside the low-energy region that is due to the 0-0 Q$_{\mathrm{y}}$ transition. The Q$_{\mathrm{x}}$ transitions would have a favorable dipole geometry to create some vibrational strength in the high-energy region. However, due to the vibronic coupling to the Q$_{\mathrm{y}}$ transition with unfavorable geometry, the rotational strength is reduced. The remaining high-energy features in the CD spectrum are removed by the inhomogeneous distribution of site energies giving rise to a localization of excited states and overlapping positive and negative bands between the different homogeneous spectra that cancel each other in the inhomogeneous spectrum.

Please note that the aspect of assuming ten modes with summed Huang–Rhys factors and averaged frequency of the 51 modes included in Reimers et al. (2013) is more critical in the case of the dimer than in the case of the monomer. Vibronic effects might be exaggerated, since coherence is maintained much longer in a basis with few stronger vibrations compared to more weaker vibrations with slightly different frequencies. While we found some changes of distinct features in the dimer spectra without inhomogeneous broadening when we included 51 modes, the differences were much less pronounced in the corresponding inhomogeneously broadened spectra (SI, Section IB).

## Conclusion

We have developed a description of absorption and CD spectra of a Chl *a* dimer in WSCP, relying on the model proposed in Reimers et al. (2013) for the Chl *a* monomer units. While we reduced the number of explicitly treated intramolecular vibrations by combining vibrational modes with similar vibrational frequencies, we extended the model by including additional modes attributed to a thermal bath. It turned out that particularly the involvement of a vibronic coupling mode is of importance for the appropriateness of the model for description of both monomer and dimer spectra. As in the monomer case investigated earlier (Reimers et al. 2013), in the absorption spectrum of the dimer we find strong signatures of the intra-monomer vibronic coupling between the 0-1 Q$_{\mathrm{y}}$ transition and the 0-0 Q$_{\mathrm{x}}$ transition. The inter-monomer excitonic couplings involving these transitions are rather weak and hidden under the inhomogeneous broadening. The latter leads to an almost complete cancelation of the CD signal outside the spectral region of the 0-0 Q$_{\mathrm{y}}$ transition. The conservative nature of the CD signal in the latter region is perturbed by the inter-monomer excitonic coupling between the 0-0 Q$_{\mathrm{y}}$ transition and the mixed 0-1 Q$_{\mathrm{y}}$/ 0-0 Q$_{\mathrm{x}}$ transitions, an effect reported here for the first time. For the present Chl *a* dimer in WSCP this perturbation is compensated by the excitonic coupling between the 0-0 Q$_{\mathrm{y}}$ and the high-energy B$_{\mathrm{x}}$ and B$_{\mathrm{y}}$ transitions. In related systems with a non-conservative CD spectrum the above compensation might be incomplete.

### Supplementary Information

Below is the link to the electronic supplementary material.

Supplementary file 1 (pdf 2062 KB)

## Appendix A Basis representation of Monomer Hamiltonian

For a representation of vibronic coupling in the basis of the respective vibrational eigenfunctions, we express the position and momentum operators of the vibronic coupling mode in terms of bosonic creation and annihilation operators $\hat{b}^{\dagger }_{\mathrm{VC}}$ and $\hat{b}_{\mathrm{VC}}$ as $\hat{q}_{\mathrm{VC}}=\sqrt{\frac{1}{2 \omega _{\mathrm{VC}}}}(\hat{b}^{\dagger }_{\mathrm{VC}}+\hat{b}_{\mathrm{VC}})$ and $\hat{p}_{\mathrm{VC}}=i \sqrt{\frac{\omega _{\mathrm{VC}}}{2}}(\hat{b}^{\dagger }_{\mathrm{VC}}-\hat{b}_{\mathrm{VC}})$. We denote the *K*-th vibrational eigenfunction of the unshifted oscillator in the electronic states $\mid \mathrm{S_1} \rangle$ and $\mid \mathrm{S_2} \rangle$ as $\mid \chi _{\mathrm{\mathrm{S_1}, VC},K} \rangle$ and $\mid \chi _{\mathrm{\mathrm{S_2}, VC},K} \rangle$, respectively, with $K \ge 0$. Using the orthogonality of the vibrational eigenfunctions, $\langle \chi _{m,\mathrm{VC},K} \mid \chi _{n,\mathrm{VC},L} \rangle =\delta _{K,L}$, and the properties of the creation and annihilation operators $\hat{b}^{\dagger }_{\mathrm{VC}} \mid \chi _{m,\mathrm{VC},L} \rangle =\sqrt{L+1} \mid \chi _{m,\mathrm{VC},L+1} \rangle$ and $\hat{b}_{\mathrm{VC}} \mid \chi _{m,\mathrm{VC},L} \rangle =\sqrt{L} \mid \chi _{m,\mathrm{VC},L-1} \rangle$, the contributions of the vibronic coupling mode to the diagonal and off-diagonal elements of the Hamiltonian represented in the electronic basis can be identified as

A1 $$\begin{aligned} \begin{aligned}&\langle \chi _{m,\mathrm{VC},K} \mid \frac{1}{2}(\omega _{\mathrm{VC}}^2 \hat{q}_{\mathrm{VC}}^2 +\hat{p}_{\mathrm{VC}}^2) \mid \chi _{m,\mathrm{VC},L} \rangle =\omega _{\mathrm{VC}} \left( K+\frac{1}{2} \right) \delta _{K,L}; \\&\quad m \in \{ \mathrm{S_1},\mathrm{S_2} \} \end{aligned} \end{aligned}$$

and

A2 $$\begin{aligned} \begin{aligned}&\langle \chi _{\mathrm{S_2},\mathrm{VC},K} \mid \alpha _{\mathrm{VC}} q_{\mathrm{VC}} \mid \chi _{\mathrm{S_1},\mathrm{VC},L} \rangle =\langle \chi _{\mathrm{S_1},\mathrm{VC},K} \mid \alpha _{\mathrm{VC}} q_{\mathrm{VC}} \mid \chi _{\mathrm{S_2},\mathrm{VC},L} \rangle \\&\quad =\alpha _{\mathrm{VC}} \sqrt{\frac{1}{2 \omega _{\mathrm{VC}}}} \left( \sqrt{K} \delta _{K-1,L}+\sqrt{K+1} \delta _{K+1,L} \right) \\&\quad =\tilde{\alpha }_{\mathrm{VC}} \sqrt{\frac{\mathrm{max}(K,L)}{2}} \delta _{\mid K-L \mid ,1}, \end{aligned} \end{aligned}$$

respectively. For the treatment of the other vibrational modes which are attributed to the system we also introduce a basis of vibrational eigenfunctions, however with respect to the shifted harmonic oscillators in the excited electronic states. We denote these vibrational eigenfunctions as $\mid \chi _{m,i,k} (d_{m,i}) \rangle$, where the electronic state to which they are assigned is specified by *m*, the index *i* refers to the selected vibrational mode and *k* is the quantum number of the selected vibrational eigenfunction. The property of the assigned harmonic oscillator to be shifted with respect to the electronic ground state is indicated by the dependence on the respective displacement $d_{m,i}$, which can formally be expressed by applying a shift operator to the corresponding vibrational eigenfunction of the unshifted oscillator in terms of $\mid \chi _{m,i,l} (d_{m,i}) \rangle =\exp (i \hat{p}_{m,i} d_{m,i}) \mid \chi _{m,i,l} \rangle$. Note that, for convenience, we keep referring to the equilibrium position of the electronic ground state as the reference position, different from Reimers et al. (2013), where the equilibrium position in S$_1$ was taken as a reference. Because of the adjustment of the basis of the vibrational eigenstates to the displacement of the harmonic oscillator, the vibrational basis representation in the subspace of each electronic state *m* becomes diagonal. The respective matrix element can be specified as

A3 $$\begin{aligned} \begin{aligned}&\langle \chi _{m,i,k} (d_{m,i}) \mid \frac{1}{2}(\omega _{i}^2 (\hat{q}_{i}-d_{m,i})^2 +\hat{p}_{i}^2) \mid \chi _{m,i,l} (d_{m,i}) \rangle \\&\quad =\omega _{i} \left( k+\frac{1}{2} \right) \delta _{k,l}. \end{aligned} \end{aligned}$$

In the vibrational basis representation of subspaces of an operator involving different electronic states non-zero matrix elements appear if the displacements of the harmonic oscillator potentials in these electronic states are different. This is the case for non-diagonal subspaces of the Hamiltonian in the electronic basis, such as $\mid \mathrm{S_2} \rangle \langle \mathrm{S_1} \mid$ and $\mid \mathrm{S_1} \rangle \langle \mathrm{S_2} \mid$ from Eq. (A1), for which the matrix elements in the basis of vibrational eigenstates of the vibronic coupling mode are given in Eq. (A2). For each pair of vibrational basis states of an intramolecular vibrational mode *i* with vibrational quantum numbers *k* and *l* in the system Hamiltonian these matrix elements are multiplied by overlap integrals $\langle \chi _{\mathrm{S_2},i,k} (d_{\mathrm{S_2},i}) \mid \chi _{\mathrm{S_1},i,l} (d_{\mathrm{S_1},i}) \rangle$ and $\langle \chi _{\mathrm{S_1},i,k} (d_{\mathrm{S_1},i}) \mid \chi _{\mathrm{S_2},i,l} (d_{\mathrm{S_2},i}) \rangle$, respectively. Also in the matrix elements of an electronic transition dipole operator $\hat{\mu }=\sum _m \mu _{m 0} \mid m \rangle \langle 0 \mid + c.c$ overlap integrals appear, so that the transition dipole strength $\mu _{m 0}$ is multiplied with $\langle \chi _{m,i,k} (d_{m,i}) \mid \chi _{0,i,l} (0) \rangle =\langle \chi _{m,i,k} (d_{m,i}) \mid \chi _{0,i,l} \rangle$, which for a non-zero displacement $d_{m,i}$ leads to non-vanishing contributions also for different quantum numbers *k* and *l*, whereas the corresponding overlap factors of the vibronic coupling mode $\langle \chi _{m,\mathrm{VC},K} \mid \chi _{0,\mathrm{VC},L} \rangle =\delta _{K,L}$ require equal quantum numbers *K* and *L* for a non-vanishing contribution. The overlap factors of the intramolecular vibrational modes can be calculated by using the recursion schemes from Manneback (1951)

A4 $$\begin{aligned} \begin{aligned}&\langle \chi _{m,i,k+1} (d_{m,i}) \mid \chi _{n,i,l} (d_{n,i}) \rangle \\&\quad =(k+1)^{-\frac{1}{2}} \left\{ l^{\frac{1}{2}} \langle \chi _{m,i,k} (d_{m,i}) \mid \chi _{n,i,l-1} (d_{n,i}) \rangle \right. \\&\qquad \left. -\sqrt{\frac{\omega _{i}}{2}} (d_{n,i}-d_{m,i}) \langle \chi _{m,i,k} (d_{m,i}) \mid \chi _{n,i,l} (d_{n,i}) \rangle \right\} \end{aligned} \end{aligned}$$

and

A5 $$\begin{aligned} \begin{aligned}&\langle \chi _{m,i,k} (d_{m,i}) \mid \chi _{n,i,l+1} (d_{n,i}) \rangle \\&\quad =(l+1)^{-\frac{1}{2}} \left\{ k^{\frac{1}{2}} \langle \chi _{m,i,k-1} (d_{m,i}) \mid \chi _{n,i,l} (d_{n,i}) \rangle \right. \\&\qquad \left. +\sqrt{\frac{\omega _{i}}{2}} (d_{n,i}-d_{m,i}) \langle \chi _{m,i,k} (d_{m,i}) \mid \chi _{n,i,l} (d_{n,i}) \rangle \right\} , \end{aligned} \end{aligned}$$

where the absolute value of the expression $\sqrt{\frac{\omega _{i}}{2}} (d_{n,i}-d_{m,i})$ can be identified with the square root of a Huang–Rhys factor $S_{mn,i}$, which in the case that *m* is the electronic ground state results as $S_{n,i}=S_{n0,i}=S_{nm,i}=\frac{1}{2} \omega _{i} d_{n,i}^2$, because of $d_{m,i}=d_{0,i}=0$. If a vibrational quantum number *k* or *l* in the recursion scheme becomes equal to zero, no further recursion steps are taken with respect to this vibrational quantum number. In the case of $l=0$ the right hand side of Eq. (A4) and (A5) is zero, anyway. By using the recursion, any overlap integral can be drawn back to the overlap between the lowest vibrational eigenfunctions, $\langle \chi _{m,i,0} (d_{m,i}) \mid \chi _{n,i,0} (d_{n,i}) \rangle =\exp \left( -\frac{S_{nm,i}}{2} \right)$. Therefore, the factor $\exp \left( -\frac{S_{nm,i}}{2} \right)$ also appears in overlap factors involving higher vibrational eigenfunctions. It accounts for the normalization of the vector of basis states and can be adjusted in view of reducing the dimension of the basis of vibrational eigenstates to an extent which still facilitates sufficiently accurate description of relevant aspects of the vibrational dynamics (in our case the vibrational features in a selected region of the absorption spectrum are of interest, as we will discuss later). In principle, the basis states with respect to the $N_{n,\mathrm{explicit}}$ intramolecular vibrational modes which are explicitly taken into account in a selected state *n* of the system Hamiltonian consist of a product of the vibrational eigenfunctions with index *i* involving all possible combinations of vibrational quantum numbers $k_i$. We specify a vector $\varvec{l}$ of dimension $N_{l,\mathrm{explicit}}$ which contains $l_i$ as its *i*-th component to formulate a single product state in terms of $\mid \phi _{n,\varvec{l}} \rangle =\prod _j \mid \chi _{n,j,l_j} (d_{n,j}) \rangle$. The vibrational basis in the subspace of state *n* can be expressed as a vector of such product states $\mid \varvec{\phi }_{n,\varvec{l}} \rangle$, where each vector component is a product state with a specific combination of vibrational quantum numbers. For the normalization we determine the overlap of the product state of the lowest vibrational eigenfunctions in electronic state *m*, namely $\mid \phi _{m,\varvec{0}} \rangle =\prod _i \mid \chi _{m,i,0} \rangle$, with each vector component of $\mid \varvec{\phi }_{n,\varvec{l}} \rangle$. If we, for example, restrict our basis to product states of the lowest vibrational eigenfunctions, except for a single vibrational eigenfunction at most, the norm of the resulting vector can be determined by introducing normalization factors $\kappa _{mn,i}$ instead of $\exp \left( -\frac{S_{mn,i}}{2} \right)$ in the overlap factors $\langle \chi _{m,i,0}(d_{m,i}) \mid \chi _{n,j,0} (d_{n,j}) \rangle =\kappa _{mn,i} \delta _{ij}$ and $\langle \chi _{m,i,0} (d_{m,i}) \mid \chi _{n,i,1} (d_{n,i}) \rangle =\sqrt{S_{mn,i}} \kappa _{mn,i}$. The condition for normalization turns out to be $\prod _i \kappa _{mn,i}^2 \sqrt{1+\sum _i S_{mn,i}}=1$. Thus, if we identify the normalization constant $\kappa _{mn}$ of the vector of the overlap factors with the product of the normalization constants of the individual modes $\prod _i \kappa _{mn,i}$, we obtain $\kappa _{mn}=\sqrt{\frac{1}{1+\sum _i S_{mn,i}}}$. Using this result for the normalization of the vector composed of the overlaps of $\mid \phi _{m,\varvec{k}} \rangle =\prod _i \mid \chi _{m,i,k_i} \rangle$ with each vector component of $\mid \varvec{\phi }_{n,\varvec{l}} \rangle$, the normalization factor $\kappa _{mn,i}$ is separated from the calculation of the overlaps of each explicitly treated vibrational mode, leading to $\langle \chi _{m,i,0} (d_{m,i}) \mid \chi _{n,j,0} (d_{n,i}) \rangle =1$ and $\langle \chi _{m,i,0} (d_{m,i}) \mid \chi _{n,i,1} (d_{n,i}) \rangle =\sqrt{S_{mn,i}}$.

To arrive at an analogous notation of the basis states as in Reimers et al. (2013), we introduce a product basis of electronic state $\mid m \rangle$, of the product state of the vibrational eigenfunction of the explicitly treated intramolecular vibrational modes $\mid \phi _{m,\varvec{k}} \rangle$ and of the vibrational eigenfunctions of the vibronic coupling mode $\mid \chi _{m,\mathrm{VC},K} \rangle$ in terms of $\mid \psi _{m,\varvec{k},K} \rangle =\mid m \rangle \mid \phi _{m,\varvec{k}} \rangle \mid \chi _{m,\mathrm{VC},K} \rangle$. In this product basis in the subspace of the singly excited states with $\{ m,n \} \in \{ \mathrm{S_1}, \mathrm{S_2} \}$ the matrix elements

A6 $$\begin{aligned} \begin{aligned}&\langle \psi _{m,\varvec{k},K} \mid \hat{H}_{\mathrm{M}} \mid \psi _{n,\varvec{l},L} \rangle =\delta _{m n} \delta _{\varvec{k},\varvec{l}} \delta _{K,L} \\&\quad \times \left\{ \epsilon _m +\left[ \omega _{\mathrm{VC}} \left( K+\frac{1}{2} \right) +\sum _i^{N_{m,\mathrm{explicit}}} \omega _{i} \left( k_i+\frac{1}{2} \right) \right] \left( \delta _{m \mathrm{S_1}}+\delta _{m \mathrm{S_2}} \right) \right\} \end{aligned} \end{aligned}$$

and

A7 $$\begin{aligned} \begin{aligned}&\langle \psi _{m,\varvec{k},K} \mid \hat{H}_{\mathrm{M}} \mid \psi _{n,\varvec{l},L} \rangle =(\delta _{m \mathrm{S_1}} \delta _{n \mathrm{S_2}}+\delta _{m \mathrm{S_2}} \delta _{n \mathrm{S_1}}) \\&\quad \times \tilde{\alpha }_{\mathrm{VC}} \sqrt{\frac{\mathrm{max}(K,L)}{2}} \delta _{\mid K-L \mid ,1} \prod _i^{N_{m,\mathrm{explicit}}} \langle \chi _{m,i,k_i} (d_{m,i}) \mid \chi _{n,i,l_i} (d_{n,i}) \rangle \end{aligned} \end{aligned}$$

are obtained, where max(K,L) gives the larger of the two vibrational quantum numbers.

## Appendix B Basis representation of Dimer Hamiltonian

In the general formulation we obtain the basis representation of the dimer Hamiltonian

B8 $$\begin{aligned} \begin{aligned}&\langle \psi _{m,\varvec{k},K}^{(a)} \psi _{m',\varvec{k}',K'}^{(b)} \mid \hat{H}_{\mathrm{D}} \mid \psi _{n,\varvec{l},L}^{(a)} \psi _{n',\varvec{l}',L'}^{(b)} \rangle \\&\quad =\langle \psi _{m,\varvec{k},K}^{(a)} \mid \hat{H}_{\mathrm{M}}^{(a)} \mid \psi _{n,\varvec{l},L}^{(a)} \rangle \langle \psi _{m',\varvec{k}',K'}^{(b)} \mid \psi _{n',\varvec{l}',L'}^{(b)} \rangle \\&\qquad +\langle \psi _{m,\varvec{k},K}^{(a)} \mid \psi _{n,\varvec{l},L}^{(a)} \rangle \langle \psi _{m',\varvec{k}',K'}^{(b)} \mid \hat{H}_{\mathrm{M}}^{(b)} \mid \psi _{n',\varvec{l}',L'}^{(b)} \rangle \\&\qquad +\langle \psi _{m,\varvec{k},K}^{(a)} \psi _{m',\varvec{k}',K'}^{(b)} \mid \hat{J} \mid \psi _{n,\varvec{l},L}^{(a)} \psi _{n',\varvec{l}',L'}^{(b)} \rangle . \end{aligned} \end{aligned}$$

The first term on the right hand side of Eq. (B8) results as

B9 $$\begin{aligned} \begin{aligned}&\langle \psi _{m,\varvec{k},K}^{(a)} \mid \hat{H}_{\mathrm{M}}^{(a)} \mid \psi _{n,\varvec{l},L}^{(a)} \rangle \langle \psi _{m',\varvec{k}',K'}^{(b)} \mid \psi _{n',\varvec{l}',L'}^{(b)} \rangle \\&\quad =\langle \psi _{m,\varvec{k},K}^{(a)} \mid \hat{H}_{\mathrm{M}}^{(a)} \mid \psi _{n,\varvec{l},L}^{(a)} \rangle \delta _{m' 0} \delta _{n' 0} \prod _i \delta _{k'_i 0} \delta _{l'_i 0} \delta _{K' 0} \delta _{L' 0}, \end{aligned} \end{aligned}$$

where $\prod _i \delta _{k'_i 0} \delta _{l'_i 0} \delta _{K' 0} \delta _{L' 0}$ appears in the evaluation of the overlap of the basis states $\langle \psi _{m',\varvec{k}',K'}^{(b)} \mid \psi _{n',\varvec{l}',L'}^{(b)} \rangle$ in combination with $\delta _{m' 0} \delta _{n' 0}$ because of the OPA. Terms containing overlaps of basis states assigned to other electronic states than the ground state vanish because due to the construction of the dimer basis such terms are multiplied with matrix elements $\langle \psi _{m,\varvec{k},K}^{(a)} \mid \hat{H}_{\mathrm{M}}^{(a)} \mid \psi _{n,\varvec{l},L}^{(a)} \rangle$, which are equal to zero for indices *m* and/or *n* referring to the electronic ground state. Otherwise, if both indices refer to a singly excited state, the evaluation of Eq. (B9) can be drawn back to the evaluation of Eqs. (A6) and (A7). Likewise, for the second term from Eq. (A6) one obtains

B10 $$\begin{aligned} \begin{aligned}&\langle \psi _{m,\varvec{k},K}^{(a)} \mid \psi _{n,\varvec{l},L}^{(a)} \rangle \langle \psi _{m',\varvec{k}',K'}^{(b)} \mid \hat{H}_{\mathrm{M}}^{(b)} \mid \psi _{n',\varvec{l}',L'}^{(b)} \rangle \\&\quad =\delta _{m 0} \delta _{n 0} \prod _i \delta _{k_i 0} \delta _{l_i 0} \delta _{K 0} \delta _{L 0} \langle \psi _{m',\varvec{k}',K'}^{(b)} \mid \hat{H}_{\mathrm{M}}^{(b)} \mid \psi _{n',\varvec{l}',L'}^{(b)} \rangle . \end{aligned} \end{aligned}$$

For the evaluation of the third term from Eq. (B8) the coupling contribution $\hat{J}$ from the dimer Hamiltonian given in Eq. (2) is expressed by the sum of its components $J^{(\bar{a}) (\bar{b})}_{\bar{m} \bar{n}} \mid \bar{m}^{(\bar{a})} \rangle \langle \bar{n}^{(\bar{b})} \mid$, where each component yields a contribution

B11 $$\begin{aligned} \begin{aligned}&\langle \psi _{m,\varvec{k},K}^{(a)} \psi _{m',\varvec{k}',K'}^{(b)} \mid J^{(\bar{a}) (\bar{b})}_{\bar{m} \bar{n}} \mid \bar{m}^{(\bar{a})} \rangle \mid 0^{(\bar{b})} \rangle \langle 0^{(\bar{a})} \mid \langle \bar{n}^{(\bar{b})} \mid \mid \psi _{n,\varvec{l},L}^{(a)} \psi _{n',\varvec{l}',L'}^{(b)} \rangle \\&\quad =J^{(a) (b)}_{m n'} \delta _{m \bar{m}} \delta _{a \bar{a}} \delta _{n' \bar{n}} \delta _{b \bar{b}} \\&\qquad \times \langle \phi _{m,\varvec{k}}^{(a)} \mid \langle \chi _{m,\mathrm{VC},K}^{(a)} \mid \delta _{m'^{(b)} 0} \langle \phi _{0,\varvec{0}}^{(b)} \mid \langle \chi _{0,\mathrm{VC},0}^{(b)} \mid \\&\qquad \times \delta _{n^{(a)} 0} \mid \phi _{0,\varvec{0}}^{(a)} \rangle \mid \chi _{0,\mathrm{VC},0}^{(a)} \rangle \mid \phi _{n',\varvec{l}'}^{(b)} \rangle \mid \chi _{n',\mathrm{VC},L'}^{(b)} \rangle \\&\quad =J^{(a) (b)}_{m n'} \delta _{m \bar{m}} \delta _{a \bar{a}} \delta _{n' \bar{n}} \delta _{b \bar{b}} \\&\qquad \times \kappa _{m 0}^{(a)} \prod _i \langle \chi _{m,i,k_i}^{(a)} \mid \chi _{0,i,0}^{(a)} \rangle \delta _{K^{(a)} 0} \kappa _{0 n'}^{(b)} \prod _j \langle \chi _{0,j,0}^{(b)} \mid \chi _{n',j,l_j}^{(b)} \rangle \delta _{L'^{(b)} 0}. \end{aligned} \end{aligned}$$

The matrix elements for a coupling contribution with opposite assignment of excited and de-excited monomer unit, i.e., for $J^{(\bar{b}) (\bar{a})}_{\bar{n} \bar{m}} \mid 0^{(\bar{a})} \rangle \mid \bar{n}^{(\bar{b})} \rangle \langle \bar{m}^{(\bar{a})} \mid \langle 0^{(\bar{b})} \mid$, can be obtained by taking the complex conjugate of Eq. (B8), thereby switching the bra- and ket side of the basis vectors. As a result, one finds a symmetry under reassignment of excited and de-excited monomer unit.

If a given coupling constant is associated with the excitonic coupling between 0-0 transitions, this aspect can be accounted for by division by the squared rescaling factor of the vibrational product states $\kappa ^2_{m0}$ to compensate the appearance of this factor in Eq. (B11).

The transition dipole operator of monomer unit $\bar{a}$ with a selected directional component $\xi \in \{ x,y,z \}$ is given as $\hat{\mu }^{(\bar{a})}_{\xi }=\sum _{\bar{m}} \mu ^{(\bar{a})}_{\xi ,\bar{m} 0} \mid \bar{m}^{(\bar{a})} \rangle \langle 0^{(\bar{a})} \mid + c.c$, where a selected sum component in the basis specified above leads to matrix elements

B12 $$\begin{aligned} \begin{aligned}&\langle \psi _{m,\varvec{k},K}^{(a)} \psi _{m',\varvec{k}',K'}^{(b)} \mid \mu ^{(\bar{a})}_{\xi ,\bar{m} 0} \mid \bar{m}^{(\bar{a})} \rangle \langle 0^{(\bar{a})} \mid \mid \psi _{n,\varvec{l},L}^{(a)} \psi _{n',\varvec{l}',L'}^{(b)} \rangle \\&\quad =\mu ^{(a)}_{\xi ,m 0} \delta _{a \bar{a}} \delta _{m \bar{m}} \kappa _{m 0}^{(a)} \prod _i \langle \chi _{m,i,k_i}^{(a)} \mid \chi _{0,i,0}^{(a)} \rangle \delta _{K^{(a)} 0} \\&\qquad \times \delta _{m'^{(b)} 0} \delta _{n'^{(b)} 0} \prod _j \delta _{k'^{(b)}_j l'^{(b)}_j} \delta _{K'^{(b)} L'^{(b)}} \\&\qquad +\mu ^{(b)}_{m' 0,\xi } \delta _{b \bar{a}} \delta _{m' \bar{m}} \delta _{m^{(a)} 0} \delta _{n^{(a)} 0} \prod _i \delta _{k^{(a)}_i l^{(a)}_i} \delta _{K^{(a)} L^{(a)}} \\&\qquad \times \kappa _{m' 0}^{(b)} \prod _j \langle \chi _{m',j,k_j}^{(b)} \mid \chi _{0,j,0}^{(b)} \rangle \delta _{K'^{(b)} 0}. \end{aligned} \end{aligned}$$

An exciton basis representation of operators given in the basis introduced above with localization of the excitation at a single monomer unit is obtained by applying a transformation with the matrix

B13 $$\begin{aligned} \begin{aligned}&\hat{A}=\mid 0 \rangle \langle 0 \mid +\sum _{\alpha } \sum _{a=1}^2 \sum _{b=1}^2 \sum _{n^{(a)}} \sum _{n'^{(b)}} \sum _{\varvec{l}^{(a)}} \sum _{\varvec{l}'^{(b)}} \sum _{L^{(a)}} \sum _{L'^{(b)}} (1-\delta _{a b})\\&\qquad \times (\delta _{n^{(a)} 0} \delta _{\varvec{l}^{(a)} \varvec{0}} \delta _{L^{(a)} 0} (1-\delta _{n'^{(b)} 0}) +(1-\delta _{n^{(a)} 0}) \delta _{n'^{(b)} 0} \delta _{\varvec{l}'^{(b)} \varvec{0}} \delta _{L'^{(b)} 0}) \\&\qquad \times c_{\alpha ; \psi _{n,\varvec{l},L}^{(a)} \psi _{n',\varvec{l}',L'}^{(b)}} \mid \alpha \rangle \langle \psi _{n,\varvec{l},L}^{(a)} \psi _{n',\varvec{l}',L'}^{(b)} \mid , \end{aligned} \end{aligned}$$

where the transformation coefficients $c_{\alpha ; \psi _{n,\varvec{l},L}^{(a)} \psi _{n',\varvec{l}',L'}^{(b)}}$ stem from diagonalization of the dimer Hamiltonian and the exciton states are denoted as $\mid \alpha \rangle$ without specifying how they depend on the vibrational structure of the monomer units. For example, the exciton basis representation of $\hat{\mu }^{(a)}$ introduced above is obtained via the transformation $\hat{\mu }_{\mathrm{exc},\xi }=\hat{A}^{\dagger } \sum _a \hat{\mu }^{(a)}_{\xi } \hat{A}$.

## Appendix C System-bath Hamiltonian and dissipative dynamics

The system part of the system-bath coupling contribution of mode $\hat{q}_{i}^{(a)}$ can be identified as

C14 $$\begin{aligned} \hat{Q}^{(\bar{a})}_{i}=\sum _{\bar{m} \in \{ \mathrm{S_1},\mathrm{S_2},\mathrm{S_3},\mathrm{S_4} \}} \mid \bar{m}^{(\bar{a})} \rangle \langle \bar{m}^{(\bar{a})} \mid . \end{aligned}$$

The representation of such an operator in the basis with excitation of a single monomer unit involving the eigenstates of the assigned vibrational modes is obtained in analogy to the corresponding basis representation of the dimer Hamiltonian in Eq. (B8) as

C15 $$\begin{aligned} \begin{aligned}&\langle \psi _{m,\varvec{k},K}^{(a)} \psi _{m',\varvec{k}',K'}^{(b)} \mid \hat{Q}^{(a)}_{i} \mid \psi _{n,\varvec{l},L}^{(a)} \psi _{n',\varvec{l}',L'}^{(b)} \rangle \\&\quad =\langle \psi _{m,\varvec{k},K}^{(a)} \mid \hat{Q}^{(a)}_{i} \mid \psi _{n,\varvec{l},L}^{(a)} \rangle \langle \psi _{m',\varvec{k}',K'}^{(b)} \mid \psi _{n',\varvec{l}',L'}^{(b)} \rangle \\&\quad =\sum _{m \in \{ \mathrm{S_1},\mathrm{S_2},\mathrm{S_3},\mathrm{S_4} \}} \delta _{m^{(a)} n^{(a)}} \\&\qquad \times \prod _i \delta _{k_i^{(a)} l_i^{(a)}} \delta _{K^{(a)} L^{(a)}} \delta _{m'^{(b)} 0} \delta _{n'^{(b)} 0} \prod _j \delta _{k'^{(b)}_j 0} \delta _{l'^{(b)}_j 0} \delta _{K'^{(b)} 0} \delta _{L'^{(b)} 0} \end{aligned} \end{aligned}$$

and

C16 $$\begin{aligned} \begin{aligned}&\langle \psi _{m,\varvec{k},K}^{(a)} \psi _{m',\varvec{k}',K'}^{(b)} \mid \hat{Q}^{(b)}_{i} \mid \psi _{n,\varvec{l},L}^{(a)} \psi _{n',\varvec{l}',L'}^{(b)} \rangle \\&\quad =\langle \psi _{m,\varvec{k},K}^{(a)} \mid \psi _{n,\varvec{l},L}^{(a)} \rangle \langle \psi _{m',\varvec{k}',K'}^{(b)} \mid \hat{Q}^{(b)}_{i} \mid \psi _{n',\varvec{l}',L'}^{(b)} \rangle \\&\quad =\sum _{m' \in \{ \mathrm{S_1},\mathrm{S_2},\mathrm{S_3},\mathrm{S_4} \}} \delta _{m^{(a)} 0} \delta _{n^{(a)} 0} \\&\qquad \times \prod _i \delta _{k_i^{(a)} 0} \delta _{l_i^{(a)} 0} \delta _{K^{(a)} 0} \delta _{L^{(a)} 0} \delta _{m'^{(b)} n'^{(b)}} \prod _j \delta _{k'^{(b)}_j l'^{(b)}_j} \delta _{K'^{(b)} L'^{(b)}}. \end{aligned} \end{aligned}$$

These results lead to the finding that in the given basis the subspace of the excited monomer unit is represented as a unity matrix, whereas the subspace of the de-excited monomer unit is represented by a matrix containing only elements equal to zero.

In the following we assume that all operators attributed to the system comprising the electronic states, the explicitly treated intramolecular vibrational modes and the vibronic coupling mode are already represented in the basis introduced above. For each bath mode *i* the bath contribution of the system-bath coupling, $\omega ^2_i d_{m,i} \hat{q}_{i}$, enters in a component of the correlation function. We assume that the fluctuations in different electronic states *m* and *n* are uncorrelated, leading to the correlation function

C17 $$\begin{aligned} \begin{aligned}&C_{m^{(a)} n^{(a)}}(t)=\delta _{m^{(a)} n^{(a)}} \sum _i d_{m^{(a)},i} \omega ^2_i d_{n^{(a)},i} \omega ^2_i \langle \hat{q}^{(a)}_i(t) \hat{q}^{(a)}_i \rangle \\&\quad =\delta _{m^{(a)} n^{(a)}} \sum _i 2 \lambda ^{(a)}_{m,i} \omega ^2_i \langle \hat{q}^{(a)}_i(t) \hat{q}^{(a)}_i \rangle , \end{aligned} \end{aligned}$$

where the time dependence in $\hat{q}^{(a)}_i(t)$ stems from a representation in the interaction picture with respect to the bath Hamiltonian. For the description of the frequency-dependent oscillator strength for a continuum of bath oscillators covering a selected frequency range we introduce the spectral density

C18 $$\begin{aligned} J(\omega )=\sum _{\nu =1,2} \pi \frac{s_{\nu }}{7! 2 \omega _{\nu }^4} \omega ^5 \exp \left( -\left( \frac{\omega }{\omega _{\nu }} \right) ^{\frac{1}{2}} \right), \end{aligned}$$

with parameters $s_{\nu }$ and $\omega _{\nu }$, where in comparison with the definition from Renger and Marcus (2002) a factor of $\pi \omega ^2$ is included. From the spectral density the correlation function is obtained as

C19 $$\begin{aligned} C(t)=\frac{1}{\pi } \int ^{\infty }_{0} d \omega J(\omega ) \left( \cos (\omega t) \coth \left( \frac{\omega }{2 k_B T} \right) -i \sin (\omega t) \right) . \end{aligned}$$

Aiming at a description in the framework of Redfield theory (May and Kühn 2011), we choose a representation in the exciton basis. Accordingly, the transformed system Hamiltonian $\hat{H}_{\mathrm{S, exc}}=\hat{A}^{\dagger } \hat{H}_{\mathrm{S}} \hat{A}$ corresponds to a diagonal matrix. The coherent part in the quantum master equation (QME) of the transformed system density matrix $\hat{\rho }_{\mathrm{exc}}=\hat{A}^{\dagger } \hat{\rho } \hat{A}$ is

C20 $$\begin{aligned} \left( \frac{\mathrm{{d}} \hat{\rho }_{\mathrm{exc}}}{\mathrm{{d}} t} \right) _{\mathrm{coh}}=-\frac{i}{\hbar } \left[ \hat{H}_{\mathrm{S, exc}}, \hat{\rho }_{\mathrm{exc}} \right] . \end{aligned}$$

In the formulation of the dissipative contribution to the Liouville-von-Neumann equation in the Redfield approach the operators $\hat{Q}^{(a)}_{i,\mathrm{exc}}=\hat{A}^{\dagger } \hat{Q}^{(a)}_{i} \hat{A}$ and

C21 $$\begin{aligned} \hat{\Lambda }^{(a)}_{i,\mathrm{exc}}(t)=\int ^{t}_0 \mathrm{{d}}\tau C(\tau ) \hat{Q}^{(a)}_{i,\mathrm{exc}}(-\tau ), \end{aligned}$$

with $\hat{Q}^{(a)}_{i,\mathrm{exc}}(-\tau )=\exp (i \hat{H}_{\mathrm{S, exc}}(-\tau )) \hat{Q}^{(a)}_{i,\mathrm{exc}} \exp (-i \hat{H}_{\mathrm{S, exc}}(-\tau ))$ enter. Note that, different from the definition in May and Kühn (2011), the upper integration border in Eq. (C21) is taken as variable. Otherwise, if the upper integration border is set to infinity, one obtains matrix elements corresponding to the Fourier-transformed correlation function at the difference frequencies associated with the time evolution of the respective matrix elements. The assumption of a variable upper integration border in $\hat{\Lambda }^{(a)}_{i,\mathrm{exc}}(t)$ (and in its adjoint operator) leads to a description of coherence dynamics at the same level as with the cumulant expansion technique (Mukamel 1995; Renger and Marcus 2002). In the case with variable upper integration border the dissipative contribution to the Liouville-von-Neumann equation in Markov approximation (here meant in the sense that the time integration is drawn into the definition of $\hat{\Lambda }^{(a)}_{i,\mathrm{exc}}(t)$ instead of involving a convolution with the density matrix) can then be formulated as

C22 $$\begin{aligned} \begin{aligned}&\left( \frac{\mathrm{{d}} \hat{\rho }_{\mathrm{exc}}}{\mathrm{{d}} t} \right) _{\mathrm{diss}}=-\sum _{a} \sum _i \left[ \hat{Q}^{(a)}_{i,\mathrm{exc}}(t), \hat{\Lambda }^{(a)}_{i,\mathrm{exc}}(t) \hat{\rho }_{\mathrm{exc}}(t) \right. \\&\quad \left. -\hat{\rho }_{\mathrm{exc}}(t) \hat{\Lambda }^{\dagger (a)}_{i,\mathrm{exc}}(t) \right] , \end{aligned} \end{aligned}$$

where $\hat{\Lambda }^{\dagger (a)}_{i,\mathrm{exc}}(t)$ contains a convolution with the complex conjugate correlation function. In the framework of the secular approximation the population dynamics is separated from the coherence dynamics (Pisliakov et al. 2006). By using subscript indices to denote matrix elements with respect to states $\alpha$ and $\beta$ in the exciton basis (involving the vibrational structure of the monomer units of the dimer), the rate expressions

C23 $$\begin{aligned} \begin{aligned}&\Gamma _{\mathrm{trans}, \alpha \beta }(t)= \sum _{a} \sum _i (\hat{Q}^{(a)}_{i,\mathrm{exc}})_{\alpha \beta } (\hat{\Lambda }^{\dagger (a)}_{i,\mathrm{exc}}(t))_{\beta \alpha } \\&\quad +\sum _{a} \sum _i (\hat{\Lambda }^{\dagger (a)}_{i,\mathrm{exc}}(t))_{\alpha \beta } (\hat{Q}^{(a)}_{i,\mathrm{exc}})_{\beta \alpha }, \end{aligned} \end{aligned}$$

are obtained, which enter in the rate equation of population transfer,

C24 $$\begin{aligned} \begin{aligned}&\left( \frac{\mathrm{{d}} (\hat{\rho }_{\mathrm{exc}})_{\alpha \alpha }}{\mathrm{{d}} t} \right) _{\mathrm{transfer}}=-\sum _{\beta \ne \alpha } \Gamma _{\mathrm{trans}, \alpha \beta }(t) (\hat{\rho }_{\mathrm{exc}})_{\alpha \alpha }(t) \\&\quad +\sum _{\alpha \ne \beta } \Gamma _{\mathrm{trans}, \alpha \beta }(t) (\hat{\rho }_{\mathrm{exc}})_{\beta \beta }(t), \end{aligned} \end{aligned}$$

and in those for coherence dephasing,

C25 $$\begin{aligned} \left( \frac{\mathrm{{d}} (\hat{\rho }_{\mathrm{exc}})_{\alpha \beta }}{\mathrm{{d}} t} \right) _{\mathrm{deph}}=-\Gamma _{\mathrm{deph}, \alpha \beta }(t) (\hat{\rho }_{\mathrm{exc}})_{\alpha \beta }(t). \end{aligned}$$

In detail, the rates $\Gamma _{\mathrm{deph}, \alpha \beta }(t)$ are composed of lifetime broadening contributions

C26 $$\begin{aligned} \begin{aligned}&\Gamma _{\mathrm{ltb}, \alpha \beta }(t)=\sum _{\gamma } \sum _a \sum _i (\hat{Q}^{(a)}_{i,\mathrm{exc}})_{\alpha \gamma } (\hat{\Lambda }^{(a)}_{i,\mathrm{exc}}(t))_{\gamma \alpha } \\&\quad +\sum _{\gamma } \sum _a \sum _i (\hat{\Lambda }^{\dagger (a)}_{i,\mathrm{exc}}(t))_{\beta \gamma } (\hat{Q}^{(a)}_{i,\mathrm{exc}})_{\gamma \beta }, \end{aligned} \end{aligned}$$

and pure dephasing contributions

C27 $$\begin{aligned} \begin{aligned}&\Gamma _{\mathrm{pd}, \alpha \beta }(t)=\sum _a \sum _i (\hat{Q}^{(a)}_{i,\mathrm{exc}})_{\alpha \alpha } (\hat{\Lambda }^{(a)}_{i,\mathrm{exc}}(t))_{\beta \beta } \\&\quad +\sum _a \sum _i(\hat{\Lambda }^{\dagger 
(a)}_{i,\mathrm{exc}}(t))_{\alpha \alpha } (\hat{Q}^{(a)}_{i,\mathrm{exc}})_{\beta \beta }. \end{aligned} \end{aligned}$$

## Appendix D Lineshape functions

The description of the dissipative dynamics in the framework of Redfield theory with secular approximation turned out to be equivalent to a treatment relying on the partial ordering description (POP) cumulant expansion used in Renger and Marcus (2002). The formulation of this approach involves the frequency-dependent correlation function

D28 $$\begin{aligned} \tilde{C}(\omega )=\int _0^{\infty } \mathrm{{d}}t \exp (i \omega t) C(t). \end{aligned}$$

The real part of this frequency-dependent correlation function can also be obtained directly from the spectral density and from the Bose-Einstein distribution

D29 $$\begin{aligned} n(\omega )=\frac{1}{\exp (-\frac{\hbar \omega }{k T})-1}, \end{aligned}$$

as

D30 $$\begin{aligned} \Re (\tilde{C}(\omega ))=(1+n(\omega )) J(\omega ) +n(-\omega ) J(-\omega ), \end{aligned}$$

thereby keeping in mind that different from Renger and Marcus (2002) a factor of $\pi \omega ^2$ has been taken into account in the spectral density given in Eq. (C18). This aspect also plays a role if the line-shape function

D31 $$\begin{aligned} G(t)=\frac{1}{\pi } \int ^{\infty }_{0} d \omega \frac{J(\omega )}{\omega ^2} \left( \cos (\omega t) \coth \left( \frac{\omega }{2 k_B T} \right) -i \sin (\omega t) \right) . \end{aligned}$$

is compared with the corresponding expression from Renger and Marcus (2002). The connection between this definition of the line-shape function and the definition where it is calculated from the correlation function (Mukamel 1995) as

D32 $$\begin{aligned} g(t)=\int _{0}^{t} d \tau \int _{0}^{\tau } d \tau ' C(\tau '), \end{aligned}$$

is the following: The analytical expression for the relation between the line-shape function and the spectral density, resulting from the combination of Eq. (D32) with Eq. (C19), can be expressed as

D33 $$\begin{aligned} \begin{aligned}&g(t)=\frac{1}{\pi } \int _0^{\infty } \mathrm{{d}} \omega \frac{1-\cos (\omega t)}{\omega ^2} \coth \left( \frac{\omega }{2 k_B T} \right) J(\omega ) \\&\quad +\frac{i}{\pi } \int _0^{\infty } \mathrm{{d}} \omega \frac{\sin (\omega t)-\omega t}{\omega ^2} J(\omega ). \end{aligned} \end{aligned}$$

One can easily convince oneself that the real part of the expression from Eq. (D33) corresponds to the difference between $\Re (G(0))$ and $\Re (G(t))$. The imaginary part from Eq. (D33) corresponds to the difference between $-i \lambda t$ and $\Im (G(t))$, where the reorganization energy $\lambda$ is identified as

D34 $$\begin{aligned} \lambda =\frac{1}{\pi } \int _0^{\infty } \mathrm{{d}} \omega \frac{1}{\omega } J(\omega ). \end{aligned}$$

Altogether one obtains

D35 $$\begin{aligned} g(t)=G(0)-G(t)-i \lambda t. \end{aligned}$$

Due to the transformation of the system component of the system-bath coupling contributions products of transformation coefficients

D36 $$\begin{aligned} \begin{aligned}&\gamma _{\nu _1 \nu _2 \nu _3 \nu _4}=\sum _{a=1}^2 \sum _{b=1}^2 \sum _{n^{(a)}} \sum _{n'^{(b)}} \sum _{\varvec{l}^{(a)}} \sum _{\varvec{l}'^{(b)}} \sum _{L^{(a)}} \sum _{L'^{(b)}} \\&\quad c_{\nu _1; \psi _{n,\varvec{l},L}^{(a)} \psi _{0,\varvec{0},0}^{(b)}} c_{\nu _2; \psi _{n,\varvec{l},L}^{(a)} \psi _{0,\varvec{0},0}^{(b)}} c_{\nu _3; \psi _{0,\varvec{0},0}^{(a)} \psi _{n',\varvec{l}',L'}^{(b)}} c_{\nu _4; \psi _{0,\varvec{0},0}^{(a)} \psi _{n',\varvec{l}',L'}^{(b)}}, \end{aligned} \end{aligned}$$

are multiplied with the line-shape function of the bath component, leading to

D37 $$\begin{aligned} g_{\alpha \beta }(t)=\gamma _{\alpha \alpha \beta \beta } g(t). \end{aligned}$$

This definition with appearance of an index pattern $\alpha \alpha \beta \beta$ implies that only diagonal elements of the system-bath coupling in the exciton basis are selected. Thus, they can be related to selected terms from the lifetime broadening contribution given in the context of Eq. (C25), namely to those involving only diagonal elements of operators attributed to system-bath coupling. Note that integration of the latter, resulting from time evolution of the QME, is required to obtain a line-shape function expression. According to Renger and Marcus (2002), in the treatment of the dissipative dynamics using the partial ordering prescription (POP) cumulant expansion it is justified to treat terms involving off-diagonal matrix elements of the system part of system-bath coupling contributions in a different way than terms involving the respective diagonal elements if either the condition $\gamma _{\alpha \alpha \alpha \alpha }>>\mid \gamma _{\alpha \beta \beta \alpha }\mid$ is fulfilled or if the dissipative dynamics takes place on a much longer timescale than the one given by $1/{\omega _{\alpha \beta }}$. Then the upper integration border in Eq. (C21) is replaced by infinity, leading to an evaluation of the Fourier-transformed correlation function at the frequency difference between the eigenenergies of the involved states of the system.
