# Supplementary material for: Signatures of intramolecular vibrational and vibronic Qx–Qy coupling effects in absorption and CD spectra of chlorophyll dimers
Source: Photosynth Res. 2022 Aug 30;156(1):19–37. doi: 10.1007/s11120-022-00946-3 (PMC10070234; doi:10.1007/s11120-022-00946-3)
Supplement: Supplementary file 1 — (pdf 2062 KB) [file 11120_2022_946_MOESM1_ESM.pdf]

Supporting Information: Signatures of intramolecular vibrational and vibronic Qx-Qy coupling effects in absorption and CD spectra of chlorophyll dimers

I. ADDITIONAL ASPECTS ABOUT MONOMER AND DIMER SPECTRA DISCUSSED IN THE MAIN TEXT

A. Role of vibronic coupling in the monomer absorption spectra

To investigate the role of the vibronic coupling in the monomer absorption spectrum, we consider separate Q_y and Q_x excitation and identify their contribution to the absorption spectrum obtained from combined excitation of Q_y and Q_x. The corresponding absorption spectra of the Chl *a* monomer with vibronic coupling and without inhomogeneous broadening for the cases without and with involvement of intramolecular vibrational modes are shown in Figs. 1 and 2, respectively. For separate excitation of Q_y the absorption spectra displayed as black lines are obtained, separate excitation of Q_x results in the absorption spectra displayed as red lines. Simultaneous excitation of Q_y and Q_x leads to the absorption spectra displayed as green lines. As the Q_y $0-1$ transition and the Q_x $0-0$ transition are energetically close to each other, the influence of vibronic coupling is recognizable particularly in the respective energetic region. In the case of Q_y it results in a peak progression, such that in addition to the main peak centered close to $0\,\mathrm{cm}^{-1}$ additional peaks slightly above $2000\,\mathrm{cm}^{-1}$ and below $4000\,\mathrm{cm}^{-1}$ appear. Even though the vibronic coupling mode itself does not exhibit a displacement, due to the vibronic coupling an effective displacement and, correspondingly, an effective Huang-Rhys factor appears [1]. The finding that the vibrational peaks are not equidistant can be explained by a shift of the Q_y $0-1$ transition due to the coupling to the Q_x $0-0$ transition. The influence of the vibronic coupling on the latter results in a splitting, which typically appears for a purely electronic coupling, but not in a recognizable peak progression. Obviously the properties of the vibronic coupling – as the origin of an effective displacement or an electronic splitting – depend on the energetic region where it gains importance for the states involved in the vibronic coupling. The previous findings are independent of the involvement of intramolecular vibrational modes in addition to the vibronic coupling mode. The intramolecular vibrational modes only lead to a broad side band below $2000\,\mathrm{cm}^{-1}$ in the case of Q_y and to a slight asymmetry of the peaks of Q_x.

B. Influence of combination of intramolecular vibrational modes in the Chl *a* monomer model and aspects of convergence depending on restrictions to the included basis states

As described in the main text, we recombined the 51 intramolecular vibrational modes of the Chl *a* monomer model specified in [2] in such a way that ten effective vibrational modes with Huang-Rhys factors and vibrational frequencies specified in Table 1 were obtained. Furthermore, only excitation of a single vibrational mode to its first excited vibrational eigenstate was taken into account. However, the possibility of simultaneous excitation of the vibronic coupling mode and this single vibrational mode was included in our treatment. The latter aspect makes the calculation numerically expensive. Under the assumption of four excited vibrational eigenstates of the vibronic coupling mode the dimension of the excited-state subspace in the product basis of the vibrational eigenstates is scaled by a factor of five. For an increased number of intramolecular vibrational modes such a factor makes a substantial

difference because of the nonlinear increase of the numerical effort with the size of the basis. This nonlinear scaling of the numerical effort also poses an obstacle for other convergence tests, for example with respect to the number of excited vibrational quanta of the intramolecular vibrational modes or with respect to the applicability of the one-particle approximation. However, by disregarding the simultaneous excitations of the vibronic coupling mode and the intramolecular vibrational modes such convergence tests are possible. We will therefore also refer to this simpler description in the following discussion of the convergence of our calculations. In Fig. 3 monomer spectra without inhomogeneous broadening obtained under different assumptions are shown. The black line is obtained from a description where simultaneous excitations of the vibronic coupling mode and a further vibrational mode of the ten modes specified in Table1 from the main text are taken into account, which in the remaining part of this paragraph will be referred to as case (a) and has been entered as a standard assumption in all calculations discussed in the main text and in the following sections. In the calculation of the absorption spectrum displayed as an orange line, which is almost identical with the black line, the number of included vibrational modes was increased to 51 (case (b)). The red and the green curve (cased (c) and (d)) stem from calculations without involvement of simultaneous excitations of vibronic coupling mode and the other intramolecular vibrational modes, but otherwise with the same assumptions as in case (a) and case (b), respectively. The blue curve (case (e)) resulted from a modification of case (c) with possible excitatation of two vibrational quanta of the intramolecular vibrational modes – either excitation to the second excited vibrational eigenstate of a single mode or two single excitations of different modes. It becomes recognizable that the black and orange curves clearly differ from the remaining ones, which are quite similar among each other. The latter finding is particularly pronounced when inhomogeneous broadening becomes involved, as Fig. 4 shows. In corresponding comparisons for the dimer an additional yellow dotted line is displayed, which stems from calculations where for the vibronic coupling mode also vibrational excitations in the electronic ground state were taken into account, but not for the intramolecular vibrational modes (case (f)). This treatment can be considered as an intermediate step of implementing a two-particle approximation (TPA) without extensively increasing the numerical effort. In Figs. 5 and 6 absorption and CD spectra of the dimer without inhomogeneous broadening are shown. The findings in the comparison of the absorption spectra are similar as in the monomer case. However, it is worth mentioning that case (d) (green line) differs from the other cases ((c)–(e)) in the spectral region of the vibrational side band of the Q_y transition and of the vibronic coupling between Q_x and Q_y. Surprisingly, in the corresponding CD spectra even an opposite sign of the peak structures of the green curve in this spectral region appears in comparison with the other curves. The enhanced amplitude of the sharp peak close to $1000\,cm^{-1}$ seems to indicate an enhanced lifetime, which is actually expected when fewer modes with larger Huang-Rhys factor are replaced by more modes with smaller Huang-Rhys factor. In contrast, the involvement of simultaneous excitations of the vibronic coupling mode and the other vibrational modes in case (b) seems to improve the reliability of the results: The absorption spectrum displayed as an orange line only exhibits minor differences in comparison with the black line, becoming smoother in the region of the first vibrational side band, and there is only a small difference in the amplitudes of the sharp side bands in the CD spectra displayed with black and orange line color. In particular, no surprising effects, such as a sign change of peaks in the side band region appear. For case (e) (blue line) one finds a distinct feature in the absorption band of the Q_y $0-0$ transition which does not appear in the other curves. In the CD spectra the amplitude is decreased compared to the other curves, most likely because of an even enhanced tendency of localization when more vibrational excitations are taken into account, relying on an analogous argumentation as

for the single vibrational excitations given in the main text. For case (f) (yellow line) the differences compared to case (c) are surprisingly small. Obviously the (partial) TPA only has a minor influence in the considered spectra. The corresponding dimer spectra with inhomogeneous broadening are shown in Figs. 7 and 8. Despite the differences in the homogeneous spectra, the green curve is very similar to the red curve. Obviously, the differences cancel out under the influence of inhomogeneous broadening. The consideration of case (b), for which the numerical calculations turned out to be extensively time-consuming, seems to be dispensable, as the differences between cases (a) and (b) were smaller than those between cases (c) and (d) in the homogeneous spectra and inhomogeneous broadening is expected to even enhance the similarity. The blue curve from case (e) exhibits remaining differences from the other curves, which can be drawn back to the findings from the homogeneous spectra. In particular, the amplitude of the CD spectrum is still decreased. However, when it is rescaled to the same height as the other curves, it does not substantially differ from them, as indicated by the violet curve. The yellow curve from case (f) is very similar to the red curve, as already observed in the homogeneous spectra. Altogether, we conclude that despite the differences in the homogeneous spectra the number of included intramolecular vibrations does not lead to any recognizable effects in the inhomogeneous spectra. The remaining differences when more than a single vibrational excitation of the intramolecular vibrational modes is taken into account mainly consist in a global scaling factor, which becomes irrelevant in our qualitative comparison with measured results in this work, anyway. Also the TPA, at least to the extent we implemented it, does not make a difference. The most critical aspect regarding the restrictions to the basis is to include simultaneous excitations of the vibronic coupling mode and the intramolecular vibrational modes, as we did in our study presented in the article.

C. Appearance of features from $\mathrm{B_x}$ and $\mathrm{B_y}$ in WSCP dimer spectra

In the upper and lower panel of Fig. 9 the absorption and CD spectra of the dimer at room temperature, which were discussed in the main text for different situations regarding electronic excitations and involvement of intramolecular vibrational modes and vibronic coupling mode are displayed with the corresponding line colors on an energy range which also captures the absorption bands resulting from involvement of the $\mathrm{B_x}$ and the $\mathrm{B_y}$ transition. However, these states were only taken into account in the calculation of the orange curve, which in the comparison of the absorption spectra is very similar to the blue curve, except for the energetic region where features from $\mathrm{B_x}$ and $\mathrm{B_y}$ excitation appear. As the transition dipole moments of $\mathrm{B_x}$ and $\mathrm{B_y}$ are relatively large, the peaks resulting from the respective transitions are the most intensive ones, even for an increased inhomogeneous broadening of $\mathrm{FWHM(B_x)} = \mathrm{FWHM(B_y)} = 1800\,\mathrm{cm^{-1}}$ which substantially flattens the peaks and decreases their intensity. The assumption of such a large inhomogeneous broadening was motivated by comparably broad peaks in the same energetic region of the measured spectra. However, it turned out that because of the large difference between the transition energies of $\mathrm{B_x}$ and $\mathrm{B_y}$ and those of $\mathrm{Q_x}$ and $\mathrm{Q_y}$ the inhomogeneous broadening of $\mathrm{B_x}$ and $\mathrm{B_y}$ transitions plays a minor role for the influence of $\mathrm{B_x}$ and $\mathrm{B_y}$ via excitonic coupling on the spectral features associated with $\mathrm{Q_x}$ and $\mathrm{Q_y}$.

D. WSCP dimer spectra at low temperature

In analogy to the investigation of dimer spectra at room temperature in the main text we also consider the low-temperature spectra at $T = 1.7\,\mathrm{K}$. A comparison of the measured absorption and CD spectra with the calculated results involving excitation of $\mathrm{B_x}$ and $\mathrm{B_y}$ is shown in the upper and lower panel of Fig. 10, respectively. As in the comparison of the spectra at room temperature, it turns out that the intensities of the side bands in the calculated absorption spectra are somewhat lower than those of the measured spectra, but that the main features are reproduced by the calculations. In particular, the low-energy part of the spectrum is reproduced very well. In the case of the CD spectrum there is practically no intensity in the high-energy region of the spectrum in excellent agreement with experiment. The negative peak is slightly smaller in the calculation than in the experiment.

II. INFLUENCE OF PROTEIN ENVIRONMENT IN WSCP BY MODIFICATION OF HUANG-RHYS FACTORS AND VIBRONIC COUPLING

In the following we investigate the influence of a variation of the vibronic coupling constant and of the Huang-Rhys factors of the intramolecular vibrational modes. We will first consider the influence of a variation of the vibronic coupling constant. In Fig. 11 the measured monomer absorption spectrum at room temperature (green line) is compared with calculated results for the previously assumed vibronic coupling constant of $750\,\mathrm{cm}^{-1}$ (black line) and for modified values of $1000\,\mathrm{cm}^{-1}$ (red line) and $500\,\mathrm{cm}^{-1}$ (blue line). It turns out that the larger value of the vibronic coupling leads to a redshift of the main peak from excitonically coupled $0-0$ $\mathrm{Q_y}$ transitions of the involved monomer units and to an intensity decrease of the first side band, but not of the second one, whereas the smaller value leads to a blueshift of the main peak, increases the intensity of the first side band and decreases the intensity of the second side band.

In the comparison of absorption spectra of the WSCP dimer at room temperature shown in the upper part of Fig. 12 similar tendencies as in the case of the monomer appear. The course of the curve obtained for decreased vibronic coupling becomes more similar to the measured spectrum, even though the intensities of the side bands are too low even in this case, while the increased vibronic coupling is not appropriate to improve the similarity of calculated and measured absorption spectra. In the corresponding CD-spectra shown in in the lower half of Fig. 12 a relative increase of the amplitude of the negative-signed peak in the energetic region of the $0-0$ $\mathrm{Q_y}$ transitions (and to a smaller extent also of the first side band) appears when the vibronic coupling is decreased and vice versa. A decrease of the vibronic coupling leads to a somewhat better agreement with experimental CD spectra. For Huang-Rhys factors of the intramolecular vibrational modes scaled by factors of 1.5 the dimer absorption and CD spectra at $T = 300\,\mathrm{K}$ are shown in the upper and lower half of Fig. 13, respectively. Again, in the absorption spectra an intensity increase of the first side band appears when the vibronic coupling is decreased, whereas for an increase of the vibronic coupling a slight decrease of the first side band is found. The second side band is rather diminished and flattened for decreased vibronic coupling, and for increased vibronic coupling the second side band gains intensity. In the CD spectra the amplitude of the negative-signed peak in the energetic region of the $0-0$ $\mathrm{Q_y}$ transitions is less pronounced than in the case of the unchanged Huang-Rhys factors of the intramolecular vibrational modes, otherwise the influence of a modification of the vibronic coupling is similar. These findings lead to the conclusion that the intensity difference

of the side bands between the measured spectra and the calculated ones could, at least partially, be compensated by decreasing the vibronic coupling and/or by increasing the Huang-Rhys factors of the intramolecular vibrational modes. A justification for adjustment of these parameters is their modification by the protein environment in WSCP compared to solution. Note, however, that such an adjustment would lead to a slightly non-conservative CD spectrum in the $Q_y(0,0)$ region, whereas the experimental spectrum is conservative.

III. ADJUSTMENT OF THE ORIENTATION OF THE TRANSITION DIPOLE VECTORS

Starting from orientations of the transition dipole moments where they are aligned with the difference vector of the position vectors of the nitrogen atoms at opposite sides of the Chl *a* molecules of the WSCP dimer (Q_y and B_y parallel to $N_B - N_D$ axis, Q_x and B_x parallel to $N_A - N_C$ axis), we apply rotations of the transition dipole moments in the molecular plane and investigate the influence on the linear spectra, particularly on the CD spectrum. From earlier works it is already known that for Q_y a rotation angle of -7° is appropriate [3]. We varied the rotation angle of the transition dipole moments of the remaining electronic states between -20° and 20°, which seems to be a realistic range for the deviation from the expected orientation of the transition dipole moments in the molecular plane, in steps of 10°. When we investigated the influence of the rotation of the transition dipole moment of a selected transition, we kept the orientations of the transition dipole moments of the other electronic transition unchanged. We disregard B_x and B_y excitation completely at first. Rotation of the transition dipole moment of Q_x leads to the absorption and CD spectra displayed in Figs. 14 and 15, respectively, together with the measured absorption spectra of the WSCP dimer from [4] in red and green line color. From calculations with rotation of $\vec{\mu}_{Q_x}$ by -20°, -10°, 0°, 10° and 20° the black, blue, violet, magenta and orange curves were obtained, respectively. While the absorption spectra are almost identical, in the CD spectra differences appear, particularly in the side bands. These differences obviously only depend on the absolute value of the rotation angle, but not on its sign. Next we consider the case that also B_x and B_y excitation are taken into account without applying a rotation to their transition dipole moments and that the rotation angle of the transition dipole moment of Q_x is varied. The resulting CD spectra are displayed in Figs. 16 and 17, where in the first a broader frequency range capturing also the energetic region of B_x and B_y transitions is chosen and in the latter the frequency range of Q_x and Q_y excitation is selected. The assignment of rotational angles and line colors is analogous to Fig. 15. Different from Fig. 15, for negative-signed angles distinct sidebands are recognizable, which become smaller with increasing rotation angle and almost vanish for the largest value of 20°. As we are aiming at results which resemble the measured spectra with their flat course in the energetic region of the side bands, we assume the latter value to be most appropriate. This choice, however, leads to a decreased amplitude of the negative-signed peak in the region of the Q_y transition in contrast to the measured CD spectrum. To compensate this tendency we still have the possibility of an appropriate adjustment of the rotation angles of the transition dipole moment of B_y and B_x, which have a smaller influence on the intensities of the vibrational sidebands than the rotation angle of $\vec{\mu}_{Q_x}$. The CD spectra for different rotation angles of $\vec{\mu}_{B_y}$ and fixed transition dipole moments $\vec{\mu}_{Q_x}$ and $\vec{\mu}_{B_x}$ are displayed in Figs. 18 and 19 on analogous frequency ranges as in Figs. 16 and 17. For the dependence of the relative amplitude of the negative-signed peak in the energetic region of Q_y on the rotation angle of $\vec{\mu}_{B_y}$ the opposite tendency than in the case of $\vec{\mu}_{Q_x}$ is found: It is increased when the rotation angle is decreased. Therefore, in view of compensating the amplitude decrease resulting from a simultaneous adjustment of the rotation angle of $\vec{\mu}_{Q_x}$, we

choose a rotation angle of -20° for the transition dipole moment of $\vec{\mu}_{\mathrm{B_y}}$. For variation of the rotation angle of $\vec{\mu}_{\mathrm{B_x}}$ the resulting CD spectra are shown in Figs. 20 and 21 in analogy to the previously discussed case of the $\mathrm{B_y}$ transition. It becomes recognizable that for an increase of the relative amplitude of the negative-signed peak in the energetic region of $\mathrm{Q_y}$ an opposite rotation of $\vec{\mu}_{\mathrm{B_x}}$ compared to $\vec{\mu}_{\mathrm{B_y}}$ is required, so that a rotation angle of 20° seems to be appropriate. The adjustment of the rotation angles of $\vec{\mu}_{\mathrm{B_x}}$ and $\vec{\mu}_{\mathrm{B_y}}$ to -20° and 20°, respectively, leads to analogous signs of the bands in the energetic region of $\mathrm{B_x}$ and $\mathrm{B_y}$ as in the measured spectra, confirming the plausibility of assuming these rotation angles. Overall these changes are not so dramatic. Assuming standard orientations for $\vec{\mu}_{\mathrm{Q_x}}$, $\vec{\mu}_{\mathrm{B_x}}$ and $\vec{\mu}_{\mathrm{B_y}}$, i.e. the x-axis for the former two and the y-axis for the latter, gives the CD spectrum in Fig. 22, which is still in good semi-quantitative agreement with the experimental data.

---

[1] J. Seibt and T. Mančal; Chem. Phys. **515** 129 (2018).

[2] J. R. Reimers, Z.-L. Cai, R. Kobayashi, M. Rätsep, A. Freiberg and E. Krausz; Sci. Rep. **3** 2761 (2013).

[3] T. Renger, M. E. Madjet, F. Müh, I. Trostmann, F.-J. Schmitt, C. Theiss, H. Paulsen, H.-J. Eichler, A. Knorr and G. Renger; J. Phys. Chem. B **113** 9948 (2009).

[4] D. M. Palm, A. Agostini, S. Tenzer, B. M. Gloeckle, M. Werwie, D. Carbonera and H. Paulsen; Biochemistry **56** 1726 (2017).

[5] J. L. Hughes, R. Razeghifard, M. Logue, A. Oakley, T. Wydrzynski and E. Krausz; J. Am. Chem. Soc. USA **128** 3649 (2006).

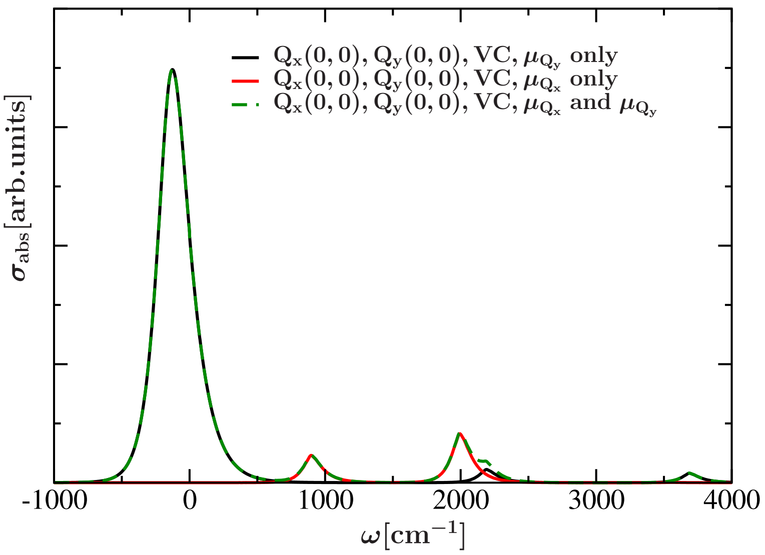

FIG. 1: Absorption spectra of Chl a monomer at $T = 300\,K$ without intramolecular vibrations, with vibronic coupling and without inhomogeneous broadening; black line: separate excitation of Q_y; red line: separate excitation of Q_x; green line: simultaneous excitation of Q_y and Q_x.

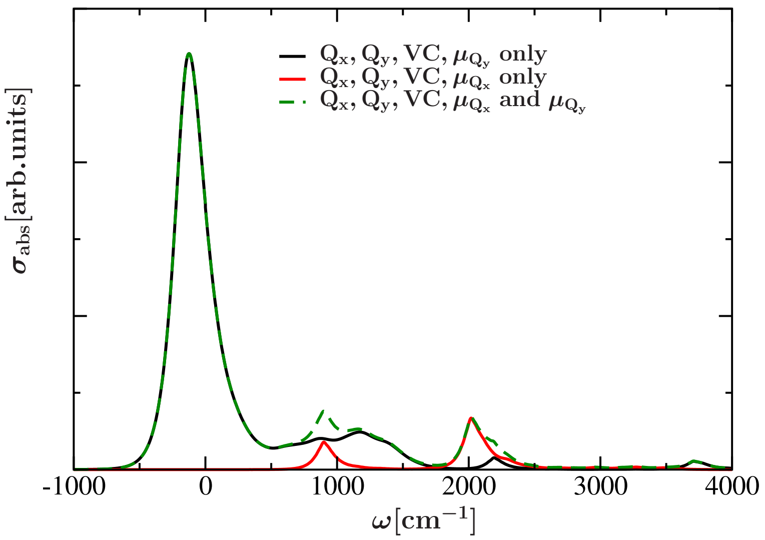

FIG. 2: Absorption spectra of Chl a monomer at $T = 300\,K$ with intramolecular vibrations, with vibronic coupling and without inhomogeneous broadening; black line: separate excitation of Q_y; red line: separate excitation of Q_x; green line: simultaneous excitation of Q_y and Q_x.

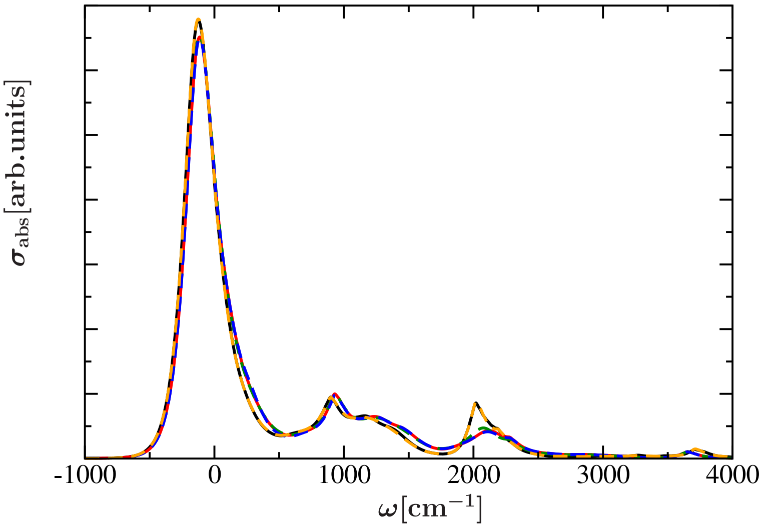

FIG. 3: Absorption spectra of monomer at $T = 300\,\mathrm{K}$ without inhomogeneous broadening; simultaneous excitation of vibronic coupling mode and of a single intramolecular vibrational mode to its first excited vibrational eigenstate was only taken into account in the cases of the spectra displayed as black and orange lines (obtained under the assumption of involvement of ten vibrational modes and of all 51 modes from [2], respectively); in all other calculations simultaneous excitation of vibronic coupling mode and another vibrational mode were disregarded; red line: assumption of possible excitation of a single intramolecular vibrational mode to its first excited vibrational eigenstate; green line: same assumption, but with involvement of all 51 modes from [2]; blue line: assumption of possible excitation of altogether two vibrational quanta among the vibrational eigenstates of the intramolecular vibrational modes.

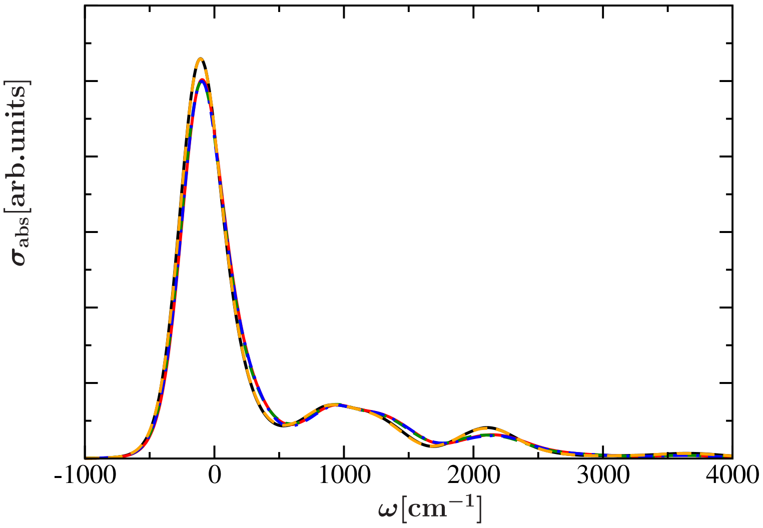

FIG. 4: Absorption spectra of monomer at $T = 300\,\mathrm{K}$ with inhomogeneous broadening; for assignment of the curves,see Fig. 3.

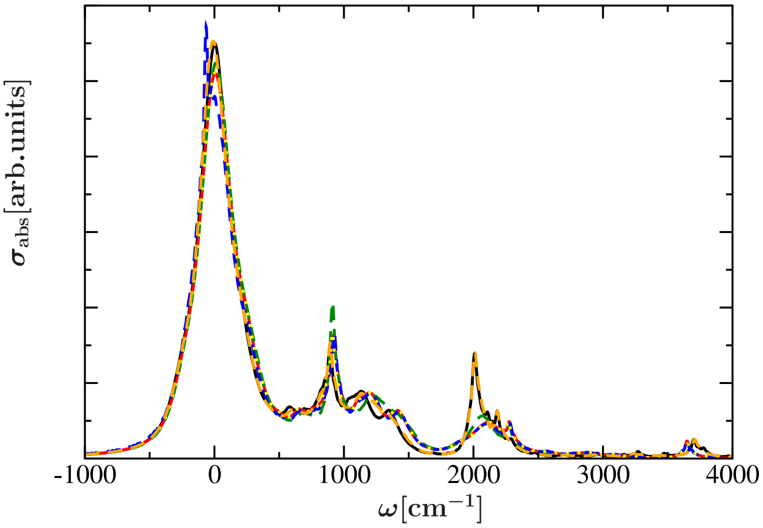

FIG. 5: Absorption spectra of dimer at $T = 300\,\mathrm{K}$ without inhomogeneous broadening and without involvement of B_x and B_x; for assignment of the lines, see Fig. 3; in addition, a yellow line is shown, which stems from a calculation with application of a TPA for the vibronic coupling mode and otherwise the same assumptions as in the calculation of the red curve.

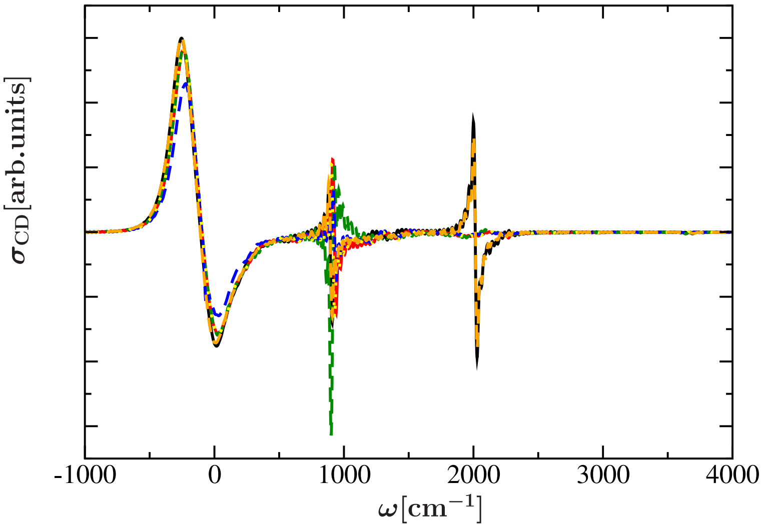

FIG. 6: CD spectra of dimer at $T = 300\,\mathrm{K}$ without inhomogeneous broadening and without involvement of B_x and B_x; for assignment of the lines, see Fig. 5.

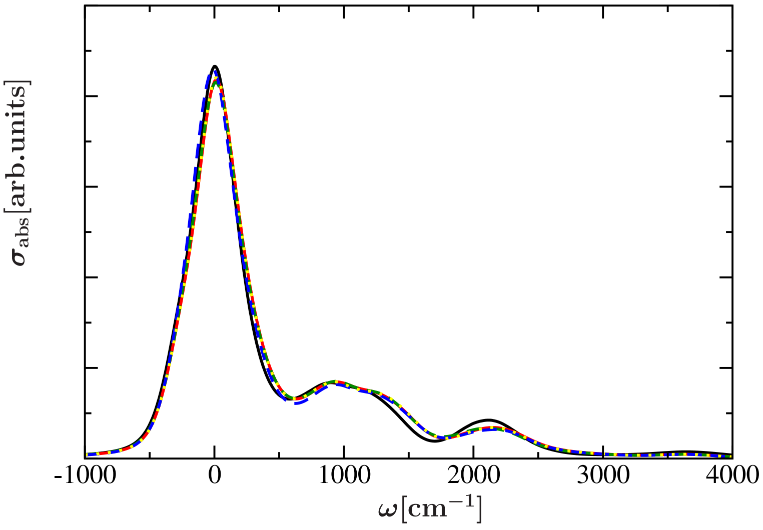

FIG. 7: Absorption spectra of dimer at $T = 300\,\mathrm{K}$ with inhomogeneous broadening and without involvement of $\mathrm{B_x}$ and $\mathrm{B_x}$; for assignment of the lines, see Fig. 5.

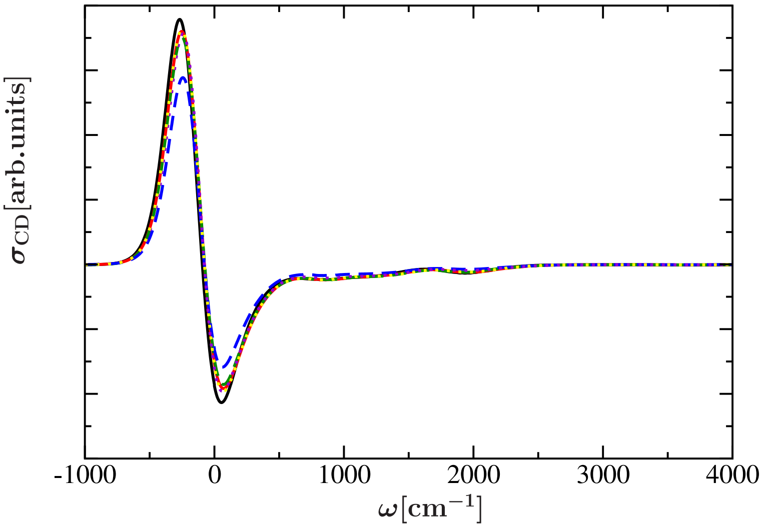

FIG. 8: CD spectra of dimer at $T = 300\,\mathrm{K}$ with inhomogeneous broadening and without involvement of $\mathrm{B_x}$ and $\mathrm{B_x}$; for assignment of the lines, see Fig. 5; for better comparability with the other curves, the spectrum displayed as a blue line is rescaled, and the result is displayed as a violet line.

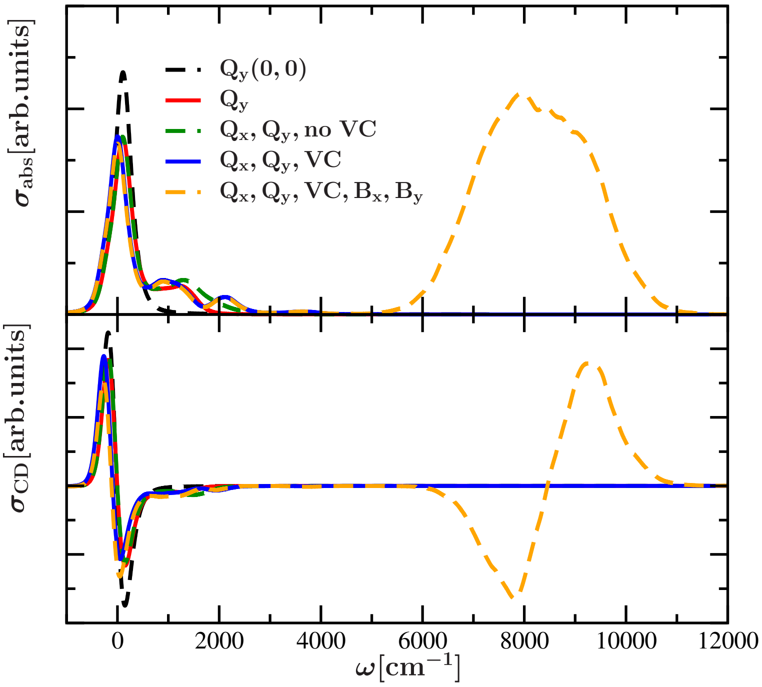

FIG. 9: Absorption and CD spectra of dimer at $T = 300\,K$ with inhomogeneous broadening, displayed over a frequency range including excitation of B_x and B_y (with FWHM(B_y) = FWHM(B_x) = $1800\,cm^{-1}$); black line: without intramolecular vibrations, excitation of Q_y only; red line: with intramolecular vibrations, excitation of Q_y only; green line: with intramolecular vibrations, excitation of Q_y and Q_x; blue line: with additional vibronic coupling; orange line: with additional involvement of B_x and B_y.

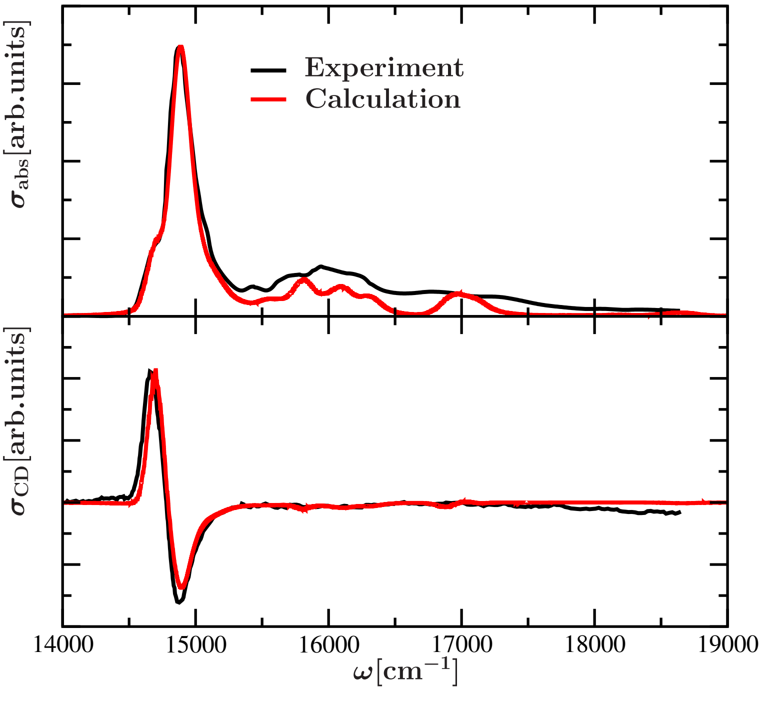

FIG. 10: Absorption spectra and CD spectra of WSCP at $T = 1.7\,K$, measured by [5] (black lines), are compared with the calculated spectra (red lines); the electronic excitation energy in the calculation was adjusted accordingly. The electronic excitation energy has been adjusted to $14920\,cm^{-1}$.

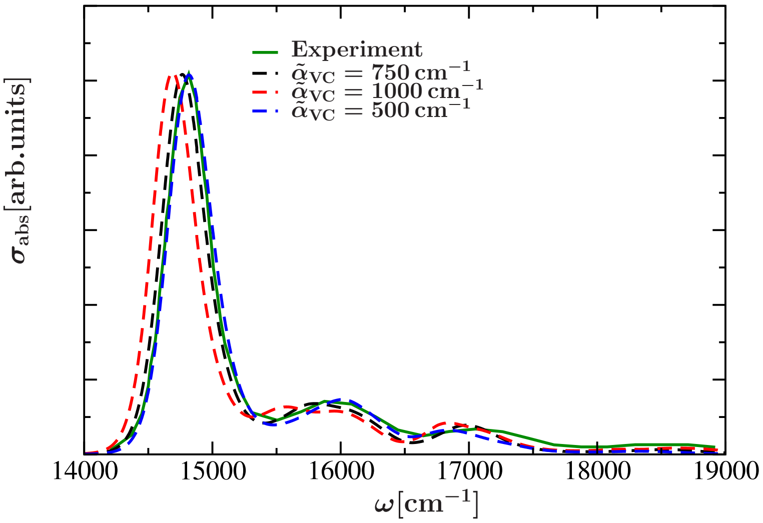

FIG. 11: Absorption spectra of Chl a in ether at $T = 300\,\mathrm{K}$: comparison of measured result [2] (green line) with calculated ones, where the vibronic coupling constant has been chosen as $750\,\mathrm{cm}^{-1}$, $1000\,\mathrm{cm}^{-1}$ and $500\,\mathrm{cm}^{-1}$, respectively, resulting in the black, red and blue line. The peaks were adjusted to a common maximum of the zero phonon line.

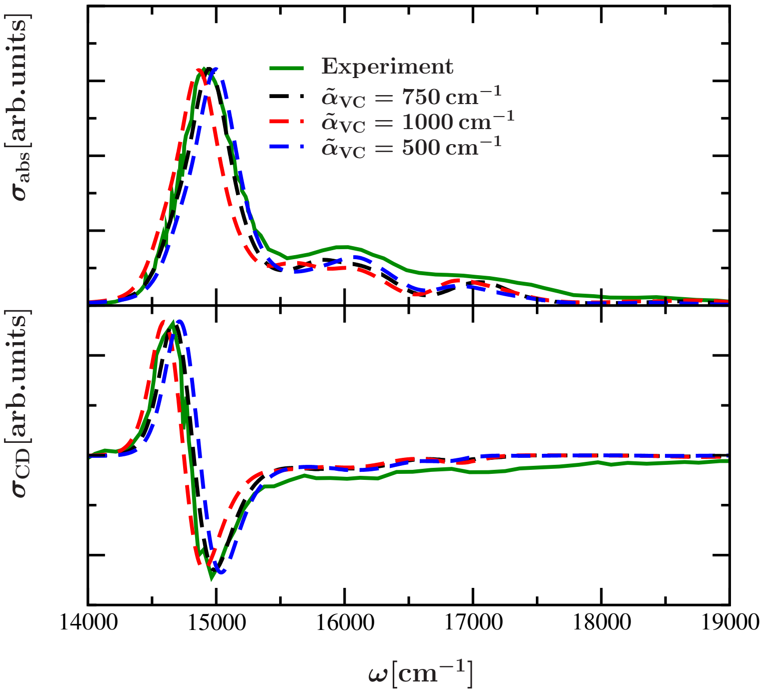

FIG. 12: Calculated absorption (upper part) and CD (lower part) spectra of dimer at $T = 300\,\mathrm{K}$ are compared with experimental data from [4]. In the calculations the vibronic coupling strength was varied as described in the legend.

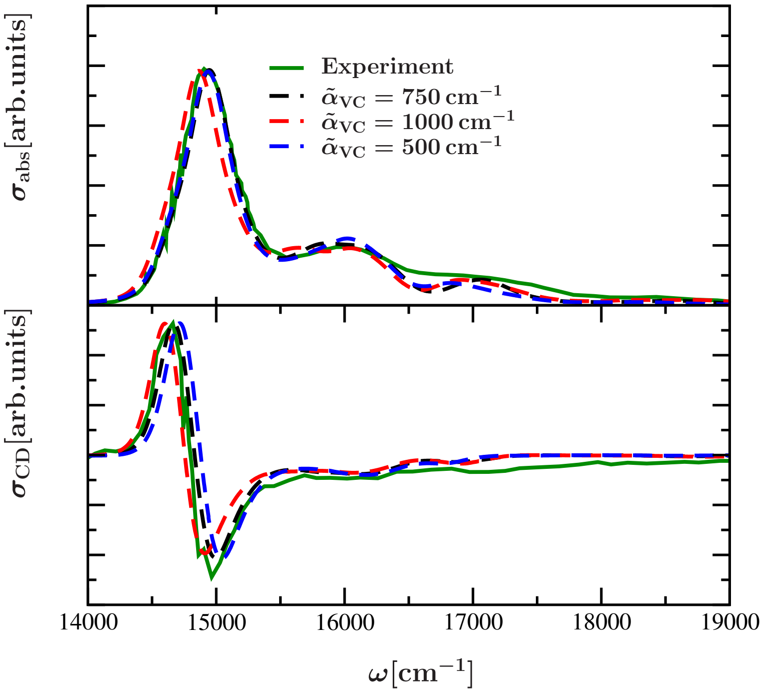

FIG. 13: Same as in Fig. 12, but in the calculations the Huang-Rhys factors of the intramolecular modes were scaled by a factor of 1.5 .

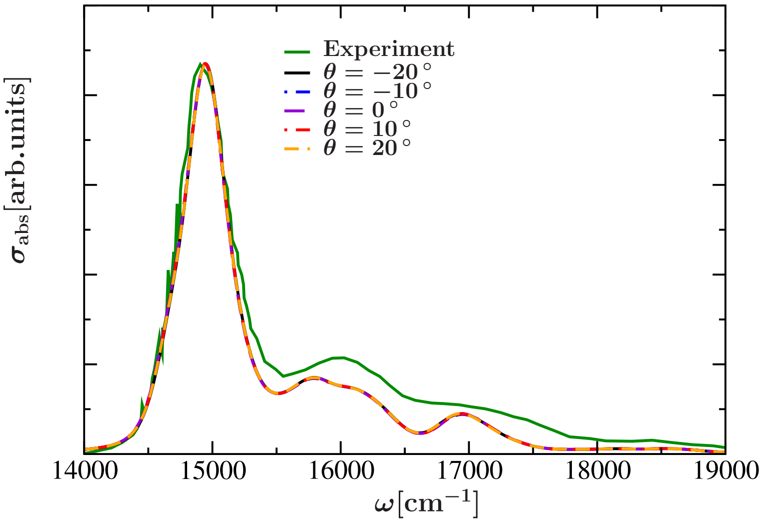

FIG. 14: Measured absorption spectrum at $T = 300\,{\rm K}$ from Palm et al., Biochemistry (2017) (green line) is displayed together with calculated absorption spectra. In the calculations a rotation of $\vec{\mu}_{\rm Q_y}$ by -7° was applied. Excitation of $\rm B_x$ and $\rm B_y$ was disregarded. For a rotation of $\vec{\mu}_{\rm Q_x}$ by -20°, -10°, 0°, 10° and 20° the black, blue, violet, red and orange curve was obtained, respectively.

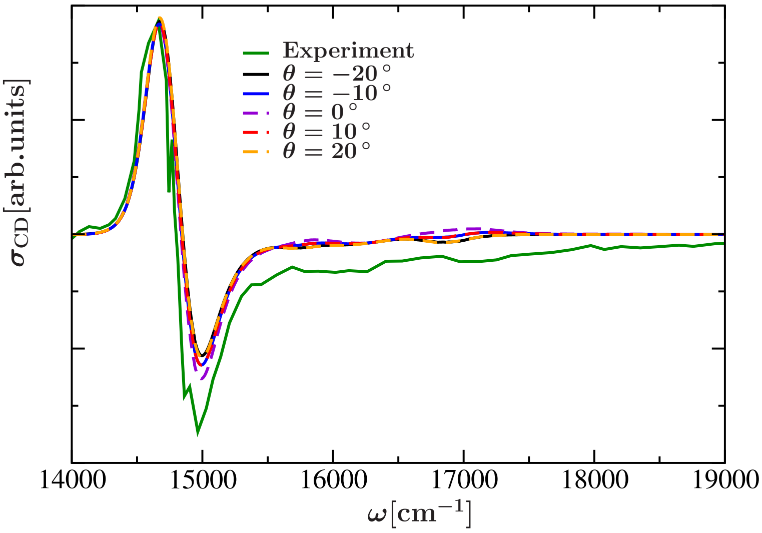

FIG. 15: Measured CD spectrum at $T = 300\,\mathrm{K}$ from Palm et al., Biochemistry (2017) (green line) is displayed together with calculated CD spectra. The assignment of the line colors of the calculated CD spectra is analogous as in Fig. 14.

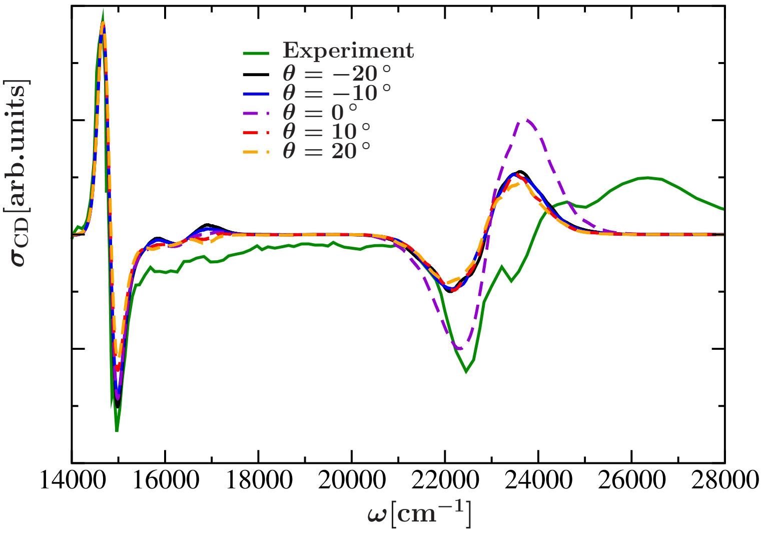

FIG. 16: Measured CD spectrum at $T = 300\,\mathrm{K}$ from Palm et al., Biochemistry (2017) (green line) is displayed together with calculated CD spectra. In the calculations a rotation of $\vec{\mu}_{\mathrm{Q_y}}$ by $-7\,^\circ$ was applied. To the transition dipole moments $\vec{\mu}_{\mathrm{B_x}}$ and $\vec{\mu}_{\mathrm{B_y}}$ no rotation was applied. For a rotation of $\vec{\mu}_{\mathrm{Q_x}}$ by $-20\,^\circ$, $-10\,^\circ$, $0\,^\circ$, $10\,^\circ$ and $20\,^\circ$ the black, blue, violet, red and orange curve was obtained, respectively.

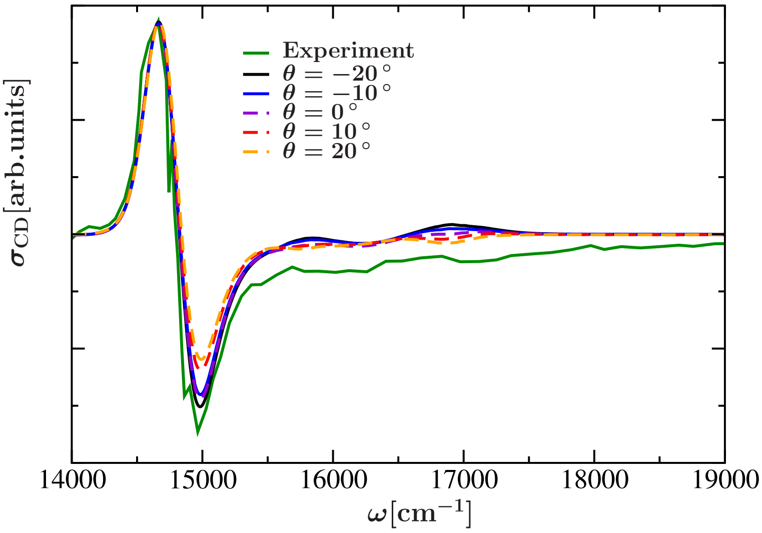

FIG. 17: The CD spectra from Fig. 16 are displayed in the energetic region of Q_x and Q_y.

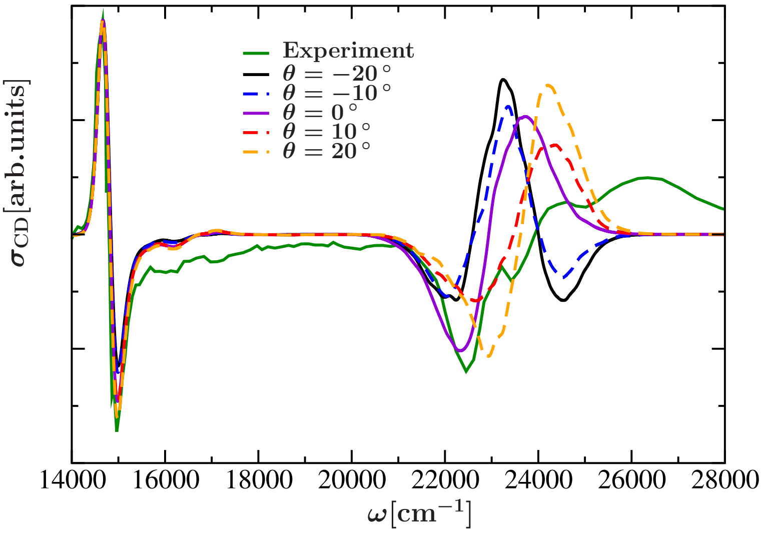

FIG. 18: Measured CD spectrum at $T = 300\,\mathrm{K}$ from Palm et al., Biochemistry (2017) (green line) is displayed together with calculated CD spectra. In the calculations a rotation of $\vec{\mu}_{\mathrm{Q_y}}$ by -7° was applied. To the transition dipole moments $\vec{\mu}_{\mathrm{Q_x}}$ and $\vec{\mu}_{\mathrm{B_x}}$ no rotation was applied. For a rotation of $\vec{\mu}_{\mathrm{B_y}}$ by -20°, -10°, 0°, 10° and 20° the black, blue, violet, ref and orange curve was obtained, respectively.

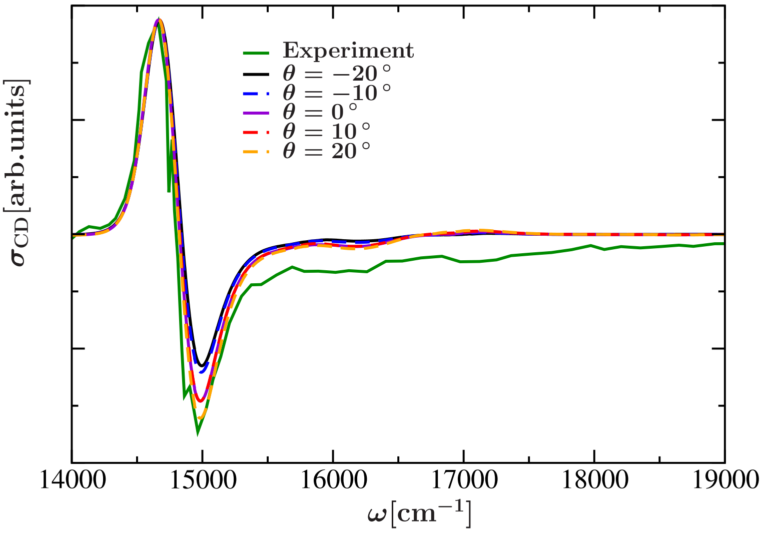

FIG. 19: The CD spectra from Fig. 18 are displayed in the energetic region of $\mathrm{Q_x}$ and $\mathrm{Q_y}$.

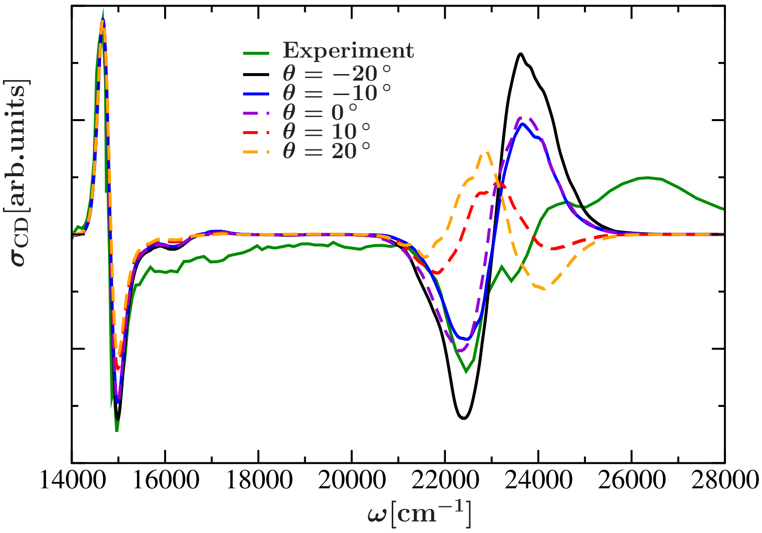

FIG. 20: Measured CD spectrum at $T = 300\,\mathrm{K}$ from Palm et al., Biochemistry (2017) (green line) is displayed together with calculated CD spectra. In the calculations a rotation of $\vec{\mu}_{\mathrm{Q_y}}$ by -7° was applied. To the transition dipole moments $\vec{\mu}_{\mathrm{Q_x}}$ and $\vec{\mu}_{\mathrm{B_y}}$ no rotation was applied. For a rotation of $\vec{\mu}_{\mathrm{B_x}}$ by -20°, -10°, 0°, 10° and 20° the black, blue, violet, red and orange curve was obtained, respectively.

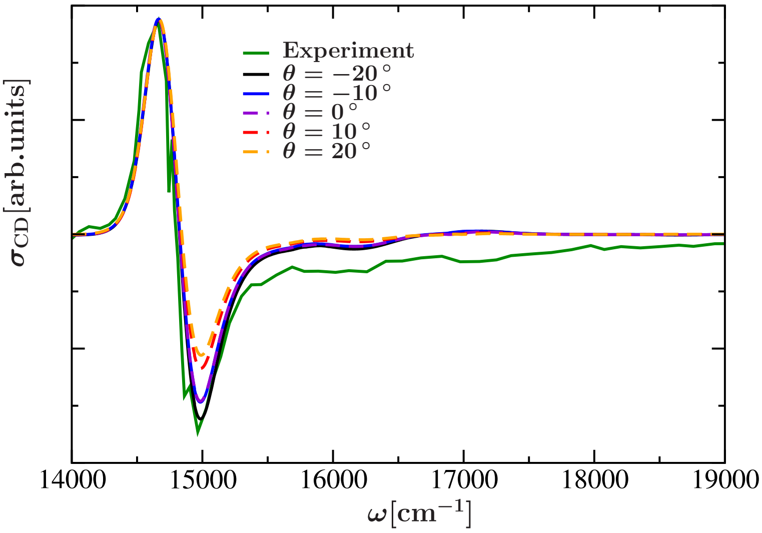

FIG. 21: The CD spectra from Fig. 20 are displayed in the energetic region of Q_x and Q_y.

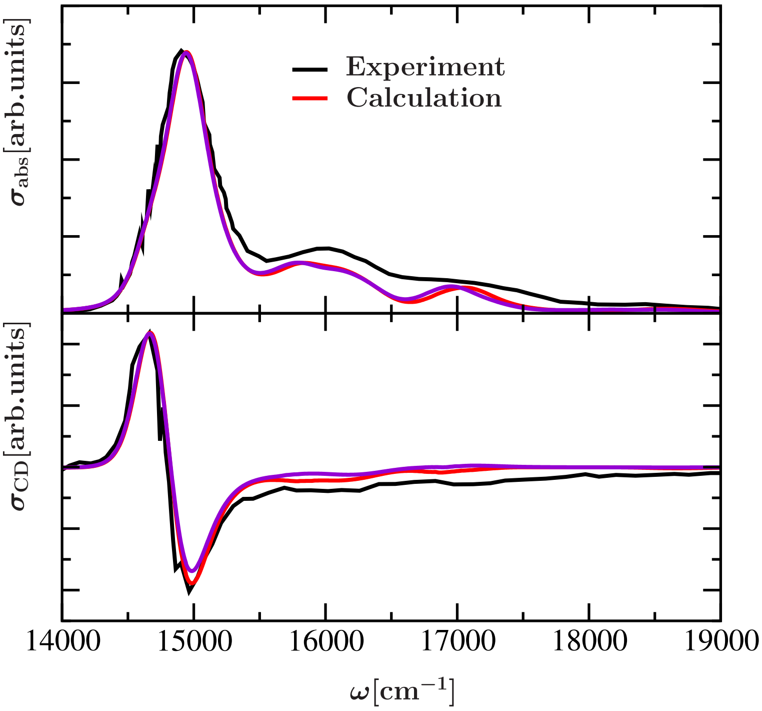

FIG. 22: Comparison between experimental (black lines) absorption (upper part) and circular dichroism (lower part) spectra of Chl *a* WSCP measured at $T = 300\,\mathrm{K}$ ([4]) and calculations in analogy to Fig. 2 from the main text. In the calculation of the red lines the rotation angles of $\vec{\mu}_{Q_x}$, $\vec{\mu}_{B_y}$ and $\vec{\mu}_{B_x}$ have been assumed as 20°, 20° and -20°, whereas in the calculation of the violet lines these rotation angles were taken as zero. In both cases a rotation by -7° has been applied to $\vec{\mu}_{Q_y}$.
